# ZNF281 inhibits mitochondrial biogenesis to facilitate metastasis of hepatocellular carcinoma

**DOI:** 10.1038/s41420-023-01691-9

**Published:** 2023-10-25

**Authors:** Qingfang Zhao, Chenguang Zhang, Xialu Zhang, Shanshan Wang, Ting Guo, Yuzhe Yin, Hui Zhang, Zhuo Li, Yang Si, Yabin Lu, Shan Cheng, Wei Ding

**Affiliations:** 1 School of Basic Medical Sciences, Capital Medical University, Beijing, 100069 China; 2 Cancer Center, Beijing Ditan Hospital, Capital Medical University, Beijing, 100015 China; 3 Beijing Institute of Hepatology, Beijing You’ An Hospital, Capital Medical University, Beijing, 100069 China; 4 Key Laboratory of Carcinogenesis and Translational Research (Ministry of Education/Beijing), Division of Gastrointestinal Cancer Translational Research Laboratory, Peking University Cancer Hospital & Institute, Beijing, 100142 China; 5 The Sixth Clinical Medical School, Capital Medical University, Beijing, 100069 China; 6 State Key Laboratory of Membrane Biology, School of Life Sciences, Tsinghua University, Beijing, 100084 China; 7 Department of Pathology, National Cancer Center/National Clinical Research Center for Cancer/Cancer Hospital, Chinese Academy of Medical Sciences and Peking Union Medical College, Beijing, 100730 China

**Keywords:** Metastasis, Metabolomics

## Abstract

Zinc finger protein 281 (ZNF281) has been shown to promote tumor progression. However, the underlying mechanism remains to be further elucidated. In this study, ZNF281 knockdown increased the expression of mitochondrial transcription factor A (TFAM) in hepatocellular carcinoma (HCC) cells, accompanied with increment of mitochondrial content, oxygen consumption rate (OCR) and levels of TCA cycle intermetabolites. Mechanistic investigation revealed that ZNF281 suppressed the transcription of TFAM, nuclear respiratory factor 1 (NRF1) and peroxisome proliferator-activated receptor γ coactivator-1α (PGC-1α). Furthermore, ZNF281 interacted with NRF1 and PGC-1α, and was recruited onto the promoter regions of TFAM, TFB1M and TFB2M repressing their expression. Knockdown of TFAM reversed ZNF281 depletion induced up-regulation of mitochondrial biogenesis and function, as well as impaired epithelial mesenchymal transition, invasion and metastasis of HCC cells. Our research uncovered a novel suppressive function of ZNF281 on mitochondrial biogenesis through inhibition of the NRF1/PGC-1α-TFAM axis, which may hold therapeutic potentials for HCC.

## Introduction

Hepatocellular carcinoma (HCC) is the most common malignancy in the liver and characterized with strong invasiveness, early recurrence, and poor prognosis [1]. There are about 700,000 deaths caused by HCC per year worldwide [2]. Therapeutic options for HCC are limited where surgical resection and liver transplantation are only suitable for about 30% of patients [3]. Although multikinase inhibitors such as sorafenib can be used in advanced HCC, drug resistance occurs quickly [4]. Clarifying molecular mechanisms of HCC will help developing therapies for this fatal disease.

ZNF281 is a member of the ZNF transcription factor family with four C2H2 zinc finger domains in structure [5]. ZNF281 specifically binds to GC-rich DNA sequences and regulates gene expression. ZNF281 orchestrates interconversion of pluripotent states in mouse embryonic stem cells (ESCs) by interaction with Ehmt1 and Zic2 [6]. Through interaction with nucleosome remodeling and deacetylation (NuRD) complex, ZNF281 participates in Nanog autorepression expression and inhibits somatic cell reprogramming [7]. ZNF281 also promotes the development of multiple types of cancer. It drives epithelial-mesenchymal transition (EMT) and metastasis in colon and breast cancer cells [8, 9]. It also promotes the progression of pancreatic cancer through augmentation of Wnt/β-catenin signaling, and neuroblastoma through inhibition of glia-derived neurotrophic factor (GDNF) and neuropilin 2 (NRP2) expression [10, 11]. We recently discovered that ZNF281 aggravates HCC through transcriptional inhibition of ANXA10 [12]. Whether ZNF281 participates in other biological processes in cancer remains elusive.

Mitochondrial biogenesis is a highly coordinated process involving finely tuned expression of nuclear and mitochondrial genes [13]. Nuclear transcription factors, such as nuclear respiratory factors 1/2 (NRF1/2), estrogen-related receptor alpha (ERRα), and peroxisome proliferator-activated receptors (PPARs), are involved in the regulation of mitochondrial biogenesis. Peroxisome proliferator-activated receptor-γ coactivator-1α (PGC-1α) interacts with these factors to dictate the expression of mitochondrial transcription factors such as mitochondrial transcription factor A (TFAM), TFB1M and TFB2M, and other nuclear-encoded mitochondrial genes. When searching for targets of ZNF281 in HCC with RNA-seq, we found significant up-regulation of TFAM upon ZNF281 silencing. TFAM regulates mitochondrial biogenesis through binding to mtDNA in sequence-specific manner to initiate the transcription of mitochondrially-encoded genes [14]. Negative regulation of ZNF281 on TFAM prompted us to study its function in mitochondrial biogenesis, which was closely related to progression of HCC.

Cancers often show altered mitochondrial biogenesis and patterns of energy production [15, 16]. In HCC, mtDNA content was decreased while the level of β-F1-ATPase was significantly lower in cancer than in normal tissue [17], suggesting suppression of mitochondrial oxidative phosphorylation and energy production. Functionally, decreased mitochondrial biogenesis is related to tumor cell progression. mtDNA deletion in colorectal cancer and prostate cancer cells can promote tumor invasion [18, 19]. Heterozygous loss of TFAM can reduce mitochondrial content and promoted the growth of intestinal tumors in mice with adenomatous polyps [20]. PGC-1α suppresses HCC progression and enhances its sensitivity to sorafenib/doxorubicin [21, 22].

In this study, we investigated the regulation of ZNF281 on mitochondrial biogenesis and function in HCC cells and explored the functional relevance between mitochondrial biogenesis with ZNF281-mediated metastasis of HCC.

## Results

### ZNF281 inhibits mitochondrial biogenesis in HCC

**Fig. 1:**
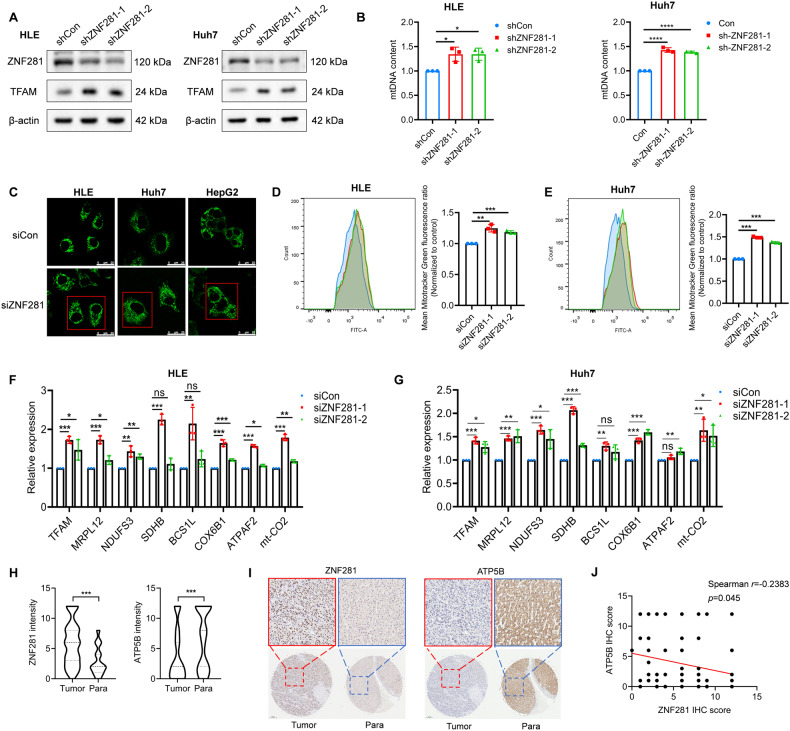
ZNF281 knockdown increases mitochondrial biogenesis in HCC cells. **A** Western blotting for TFAM following knockdown of ZNF281 in both HLE and Huh7 cells. **B** Relative mtDNA content measured by qPCR in ZNF281 knockdown HLE and Huh7 cells. **C** Confocal laser scanning microscope analyses of mitochondrial mass in HCC cells with mitotracker green staining. Scale bars, 25 μm. **D**, **E** Left: Flow cytometry analyses of mitochondrial mass alteration with mitotracker green staining in HLE or Huh7 cells; Right: statistical analyses of fluorescence. **F**, **G** The mRNA levels of TFAM and genes encoding different ETC subunits were measured by RT-qPCR in ZNF281 knockdown HLE and Huh7 cells. **H**, **I** Immunohistochemistry of ZNF281 and ATP5B in HCC tissues in comparison with corresponding para-cancerous tissues (lower: 5×, upper: 20× objective). **J** ZNF281 and ATP5B IHC score correlation was analyzed. *p < 0.05, **p < 0.01, and ***p < 0.001. All experiments were performed at least three times.

We previously knocked down ZNF281 in HLE cell lines with two different shRNAs to find its potential targets with RNA-seq [12]. The mRNA level of TFAM was significantly increased with ZNF281 knockdown (Supplementary Fig. 1A, B), as well as the protein level determined with western blot (Fig. 1A and Supplementary Fig. 2A) and immunofluorescence (Supplementary Fig. 2B) in HLE and Huh7 cells. Gene Set Enrichment Analysis (GSEA) indicated negative enrichment of “Oxidative phosphorylation” in the control group (Supplementary Fig. 1C, D), suggesting negative correlation of ZNF281 with mitochondrial biogenesis and function in HCC.

To verify the effect of ZNF281 on the mitochondrial biogenesis, we detected changes of mtDNA content with ZNF281 depletion, which significantly up-regulated mtDNA content in HLE and Huh7 cells (Fig. 1B and Supplementary Fig. 2C). Mitotracker Green staining and cellular immunofluorescence against mitochondrial proteins indicated obvious increment of mitochondria mass upon ZNF281 knockdown in HCC cells (Fig. 1C–E and Supplementary Fig. 2E). Interestingly, ZNF281 silencing in lung cancer cell A549 and colon cancer cell line HCT-8 also significantly increased mitotracker green staining (Supplementary Fig. 2D), indicating that inhibition of ZNF281 on mitochondrial biogenesis was not limited to HCC.

Effect of ZNF281 on mitochondrial gene expression was also detected. RT-qPCR showed that selected genes of different mitochondrial electron transport chain (ETC) complexes were up-regulated upon ZNF281 knockdown (Fig. 1F, G). Correlation analyses between ZNF281 and the ETC subunits at the mRNA level were performed using data from the TCGA hepatocellular carcinoma database. Results showed significant negative correlation between ZNF281 and these subunits (Supplementary Fig. 2F). Furthermore, a number of mitochondrial expressed genes showed obvious negative correlation with ZNF281 from the same database (Supplementary Table S1). These results strongly support inhibitory effect of ZNF281 on mitochondrial biogenesis in HCC. Similar correlations were also observed in TCGA colorectal adenocarcinoma and lung adenocarcinoma (Supplementary Table S2 and 3, respectively). Immunohistochemistry was then performed to determine the correlation of ZNF281 and ATP5B (ATP synthase F1 subunit beta in mitochondria) at the protein level using HCC tissue arrays. ZNF281 showed higher expression in cancer tissues than in para-cancerous tissues while ATP5B showed the opposite pattern(Fig. 1H), supporting previous reports [17]. More importantly, the level of ZNF281 negatively correlates with that of ATP5B (Fig. 1I, J). Further analysis of clinicopathological characteristics indicated that the protein level of ZNF281 was positively correlated while ATP5B negatively correlated with pathological grade of HCC patients (Supplementary Fig. 2G and Tables 1 and 2).

**Table 1:** The correlation between ZNF281 expression and clinicopathological characteristics in HCC tissue microarray (71 samples).

Variables	ZNF281 low	ZNF281 high	*P*-value
	(*n* = 16)	(*n* = 55)	
Age (years)			0.592
≤55	9	35	
>55	7	20	
Gender			0.934
Male	12	44	
Female	4	11	
Pathological grade			0.038
I	3	2	
II–III	13	53	
Tumor stage			
I	4	7	0.66
II	4	14	
III	6	28	
IV	2	6

**Table 2:** The correlation between ATP5B expression and clinicopathological characteristics in HCC tissue microarray (71 samples).

Variables	ATP5B low	ATP5B high	*P*-value
	(*n* = 44)	(*n* = 27)	
Age (years)			0.893
≤55	27	17	
>55	17	10	
Gender			0.673
Male	34	22	
Female	10	5	
Pathological grade			0.045
I	1	4	
II- III	43	23	
Tumor stage			
I	4	7	0.255
II	11	7	
III	24	10	

### ZNF281 inhibits mitochondrial function of HCC cells

**Fig. 2:**
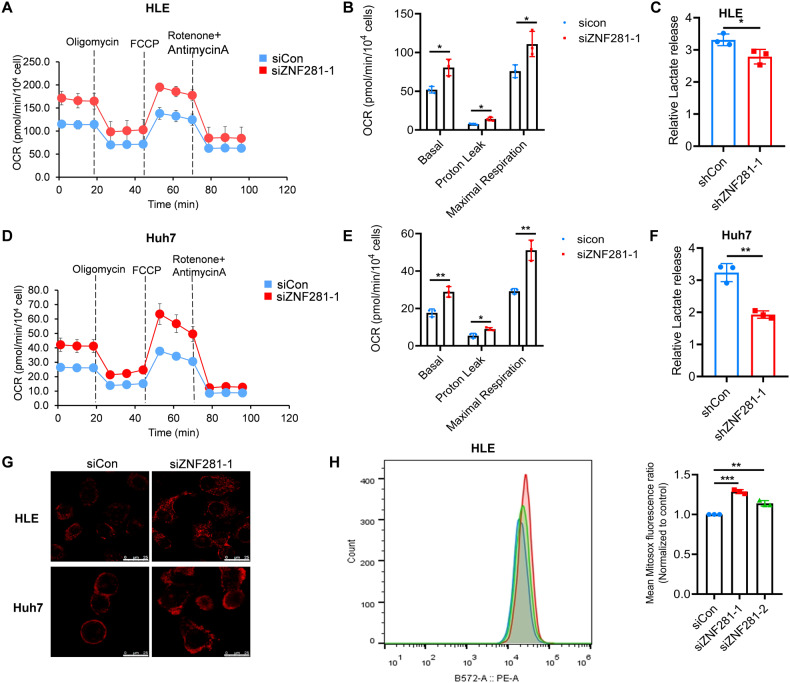
Knockdown of ZNF281 enhances mitochondrial function of HCC cells. **A** Measurement of OCR in ZNF281 knockdown HLE cells. **B** Basal respiration, proton leak, and maximal respiration from (**A**) were quantified. **C** Lactate release was determined in ZNF281 knockdown HLE cells. **D** Measurement of OCR in ZNF281 knockdown Huh7 cells. **E** Basal respiration, proton leak, and maximal respiration from (**D**) were quantified. **F** Lactate release was determined in ZNF281 knockdown Huh7 cells. **G** Confocal laser scanning microscope detected MitoSox fluorescence in HLE and Huh7 cells (bar = 25 μm). **H** Left: Flow cytometry analyses of MitoSox fluorescence in HLE or Huh7 cells; Right: statistical analysis of MitoSox fluorescence. *p < 0.05, **p < 0.01, and ***p < 0.001. All experiments were performed at least three times.

Mitochondrial function was also determined with ZNF281 knockdown. Results of oxygen consumption rate (OCR) showed that the basal oxygen consumption rate, proton leak, and maximum oxygen consumption rate were all significantly increased with ZNF281 knockdown, indicating augmentation of oxidative phosphorylation and mitochondrial function (Fig. 2A–E). Meanwhile, lactic acid release was decreased by ZNF281 depletion, indicating impaired aerobic glycolysis (Fig. 2C, F).

Oxidative phosphorylation was often accompanied with reactive oxygen species (ROS) production, MitoSox staining indicated increased mitochondrial ROS after ZNF281 knockdown (Fig. 2G, H).

**Fig. 3:**
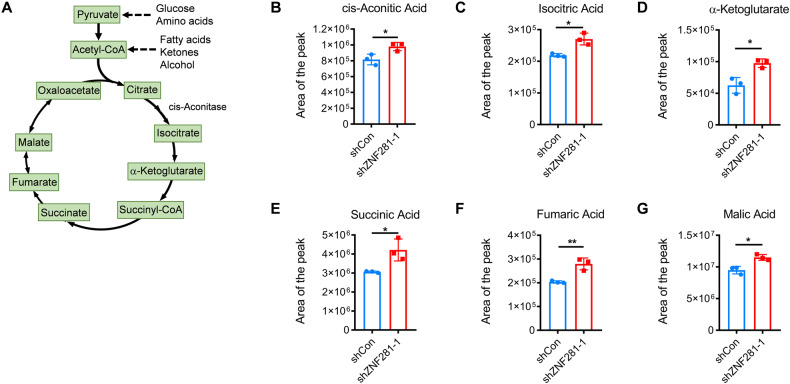
Knockdown ZNF281 increased mitochondrial TCA cycle metabolites. **A** Schematic diagram of TCA cycle. **B**–**G** The level of intermediate metabolites of TCA cycle were determined (*n* = 3; *p < 0.05, **p < 0.01).

Target metabolomics was further performed to detect changes of the intermediate metabolites of tricarboxylic acid (TCA) cycle in mitochondria. Nearly all the intermediate metabolites were increased after ZNF281 knockdown (Fig. 3A–G), suggesting augmentation of mitochondrial functions.

### ZNF281 represses the expression of NRF1 and PGC-1α in HCC cells

**Fig. 4:**
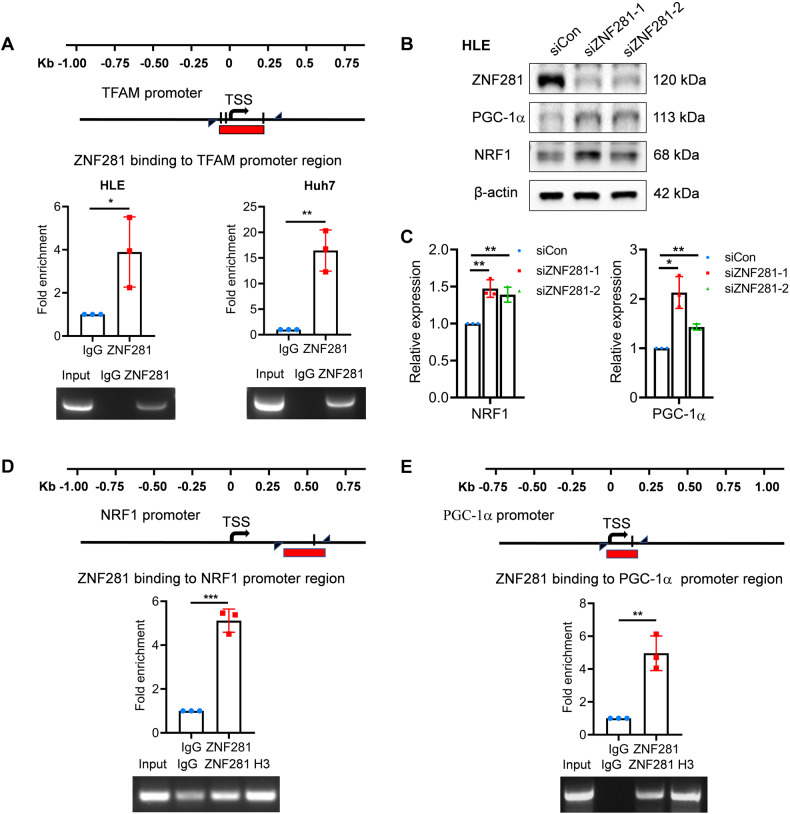
ZNF281 inhibits the expression of PGC-1α-NRF1-TFAM axis in HCC cells. **A** Upper: Scheme of TFAM promoter region containing potential ZNF281 binding motifs. Triangular arrows indicate primers. Red line represents the amplified product. Black boxes indicate GC-rich sequence. Middle: ChIP-qPCR demonstrating the enrichment of ZNF281 at TFAM promoter in HLE/Huh7 cells. Lower: Analysis of ChIP with semi-quantitative PCR and agarose gel electrophoresis. **B** Western blotting for NRF1 or PGC-1α following ZNF281 knockdown in HLE cells. **C** mRNA levels of NRF1 or PGC-1α was measured by RT-qPCR in HLE cells. **D**, **E** Upper: Scheme of NRF1 or PGC-1α promoter region containing potential ZNF281 binding motifs for ChIP analyses. Middle: ChIP-qPCR demonstrating the enrichment of ZNF281 at PGC-1α/NRF1 promoter in HLE cells. Lower: Analysis of ChIP results with semi-quantitative PCR and agarose gel electrophoresis. *p < 0.05, **p < 0.01, and ***p < 0.001. All experiments were performed at least three times.

Potential GC-rich binding motifs of ZNF281 were found around the transcription start site (TSS) of TFAM. chromatin immunoprecipitation (ChIP) showed that ZNF281 directly bind to these sequences on TFAM promoter (Fig. 4A), indicating a direct regulation. Since ZNF281 also modulated the expression of multiple mitochondrial genes (Fig. 1F, G), and some of which were not targets of TFAM, this suggest that regulation of ZNF281 on mitochondrial genes was not limited to TFAM, and might involve upstream PGC-1α/NRF1 axis. Supporting this speculation, ZNF281 knockdown resulted in significant up-regulation of PGC-1α and NRF1 at both the mRNA and protein levels (Fig. 4B, C). Potential binding motifs of ZNF281 were also found around the TSS of PGC-1α and NRF1. ChIP analyses indicated ZNF281 directly bound to these motifs (Fig. 4D, E). These results indicated that ZNF281 inhibited PGC-1α-NRF1-TFAM axis directly to suppress mitochondrial biogenesis.

### ZNF281 interacts with NRF1/PGC-1α to regulate mitochondrial transcription factors

**Fig. 5:**
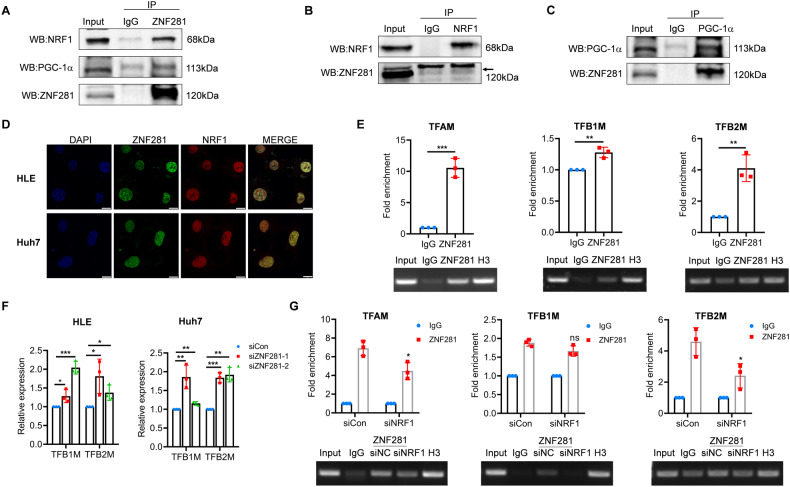
ZNF281 regulates expression of TFAM, TFB1M and TFB2M through interaction with NRF1/PGC-1α. **A**–**C** Co-immunoprecipitation (Co-IP) analyses for the interaction of ZNF281 with NRF1/PGC-1α. **D** Immunofluorescence showed co-localization of ZNF281 and NRF1 in HLE and Huh7 cells (bar = 10 μm). **E** Upper: ChIP-qPCR demonstrating the enrichment of ZNF281 in the promoter regions of TFAM/TFB1M/TFB2M harboring NRF1 binding sites. Lower: Analyses of ChIP results by semi-quantitative PCR and agarose gel electrophoresis. **F** The mRNA levels of TFB1M/TFB2M were measured by RT-qPCR with ZNF281 knockdown. **G** Upper: ChIP-qPCR demonstrating the enrichment of ZNF281 in the promoter regions of TFAM/TFB1M/TFB2M harboring NRF1 binding sites in NRF1 knockdown HLE cells. Lower: Analysis of ChIP results with semi-quantitative PCR and agarose gel electrophoresis. *p < 0.05, **p < 0.01, and ***p < 0.001. All experiments were performed at least three times.

In ESCs, ZNF281 promotes Nanog expression while simultaneously interacts with Nanog to control pluripotency [7]. We wonder if ZNF281 also interacts NRF1 and PGC-1α like in the case of Nanog. Co-IP experiments in HLE cells showed endogenous interaction of ZNF281 with NRF1 and PGC-1α (Fig. 5A–C). Immunofluorescence showed co-localization of ZNF281 and NRF1 in the nuclei (Fig. 5D), supporting their interaction. ZNF281 and NRF1 also showed co-localization in A549 and HCT-8 cells (Supplementary Fig. 3A).

Mitochondrial transcription factors TFAM, TFB1M, and TFB2M are direct targets of NRF1/ PGC-1α. We next detected if ZNF281 was recruited by NRF1 onto the promoters of TFAM, TFB1M, and TFB2M. ChIP assays showed enrichment of ZNF281 on the promoters of TFAM, TFB1M and TFB2M in the same regions as NRF1 (Fig. 5E and Supplementary Fig. 3B). Similar to TFAM, ZNF281 knockdown increased the mRNA level of TFB1M and TFB2M (Fig. 5F), suggesting general inhibition of ZNF281 on mitochondrial transcription factors.

To elucidate the dependence of NRF1 for efficient inhibition of TFAM, TFB1M, and TFB2M, NRF1 was knocked down in HLE cells, which decreased its binding in the promoters of TFAM, TFB1M and TFB2M (Supplementary Fig. 3D). Enrichment of ZNF281 in these promoter regions of TFAM and TFB2M was also reduced (Fig. 5G). These results indicated that the ZNF281 bind to the promoters of mitochondrial transcription factors together with NRF1.

Analyses of expression data in GSE14323 showed increased expression of ZNF281 in HCC compared with normal and cirrhosis liver tissues but decreased expression of PGC-1α, TFAM, TFB1M, and TFB2M (Supplementary Fig. 4). NRF1 showed a downward trend of expression in HCC compared with cirrhosis tissues. These results suggested negative regulation of ZNF281 on PGC-1α-NRF1-TFAM/TFB1M/TFB2M.

### NRF1/TFAM attenuates the inhibitory effect of ZNF281 on mitochondrial biogenesis

**Fig. 6:**
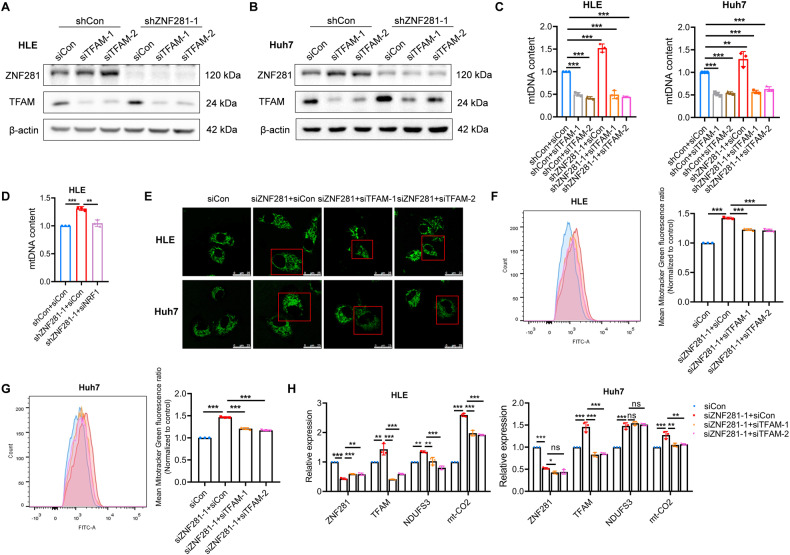
TFAM or NRF1 knockdown attenuates ZNF281 depletion-induced mitochondrial biogenesis. **A, B** Western blot analyses of ZNF281 and TFAM in HLE (**A**) and Huh7 (**B**) cells. **C** Relative mtDNA content was measured by qPCR in ZNF281 and TFAM knockdown HLE and Huh7 cells. **D** Relative mtDNA content was measured by qPCR in ZNF281 and NRF1 knockdown HLE cells. **E** Confocal laser scanning microscope analyses of mitochondrial mass in HCC cells with mitotracker green staining. Scale bars, 25 μm. **F, G** Left: Flow cytometry analyses of mitochondrial mass with mitotracker green staining in ZNF281 and TFAM knockdown HLE or Huh7 cells; Right: statistical analysis of mitotracker fluorescence. **H** mRNA levels of TFAM and genes encoding different mitochondrial ETC subunits were measured by RT-qPCR in ZNF281 and TFAM knockdown HLE and Huh7 cells. *p < 0.05, **p < 0.01, and ***p < 0.001. All experiments were performed at least three times.

The functionality of NRF1/TFAM in ZNF281-mediated inhibition of mitochondrial biogenesis was further investigated. siRNAs against TFAM abolished the increment of mtDNA content in ZNF281 knockdown HCC cells (Fig. 6A–C), which was also the case in HLE cells with simultaneous knockdown of NRF1 and ZNF281 (Fig. 6D and Supplementary Fig. 5A). Mitotracker Green staining showed that knockdown of TFAM partially reversed the increment in mitochondrial mass by ZNF281 depletion (Fig. 6E–G and Supplementary Fig. 5B, C). Up-regulation of genes encoding subunits of different ETC complexes was also reversed by simultaneous TFAM knockdown (Fig. 6H Supplementary Fig. 5D).

**Fig. 7:**
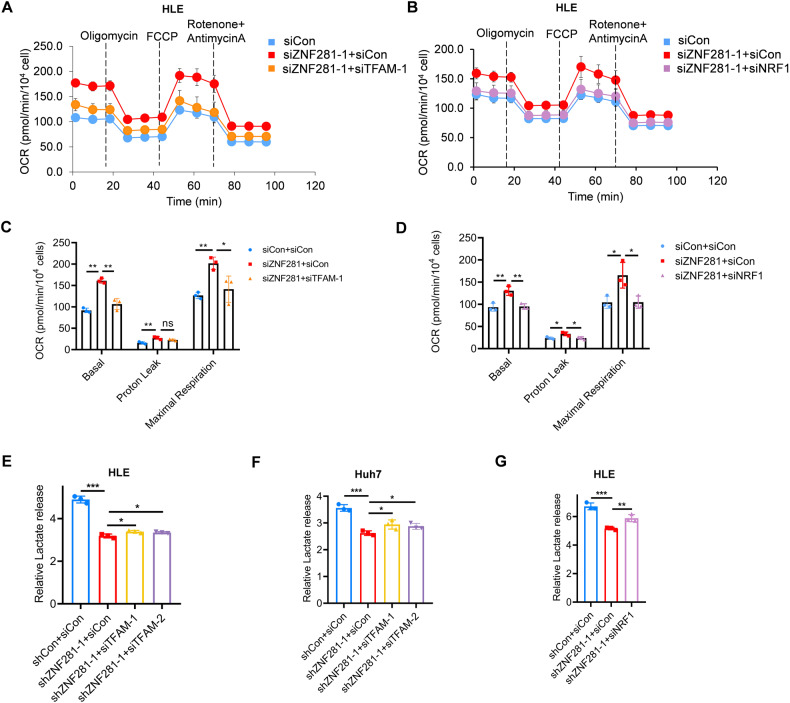
TFAM or NRF1 knockdown alleviates the effect of ZNF281 depletion on mitochondrial function. **A** Measurement of OCR in ZNF281 and TFAM knockdown HLE cells. **B** Measurement of OCR in ZNF281 and NRF1 knockdown HLE cells. **C** Basal respiration, proton leak, and maximal respiration from (**A**) were quantified. **D** Basal respiration, proton leak, and maximal respiration from (**B**) were quantified. **E, F** Lactate release was determined in ZNF281 and TFAM knockdown HCC cells. **G** Lactate release was determined in ZNF281 and NRF1 knockdown HLE cells. *p < 0.05, **p < 0.01, and ***p < 0.001. All experiments were performed at least three times.

Analyses of OCR showed that knocking down either TFAM or NRF1, partially attenuated the increased OCR levels upon ZNF281 knockdown (Fig. 7A–D). Furthermore, TFAM or NRF1 knockdown also alleviated the reduced lactate release due to ZNF281 depletion (Fig. 7E–G).

### Inhibition of mitochondrial biogenesis contributes to ZNF281-mediated HCC invasion and metastasis

**Fig. 8:**
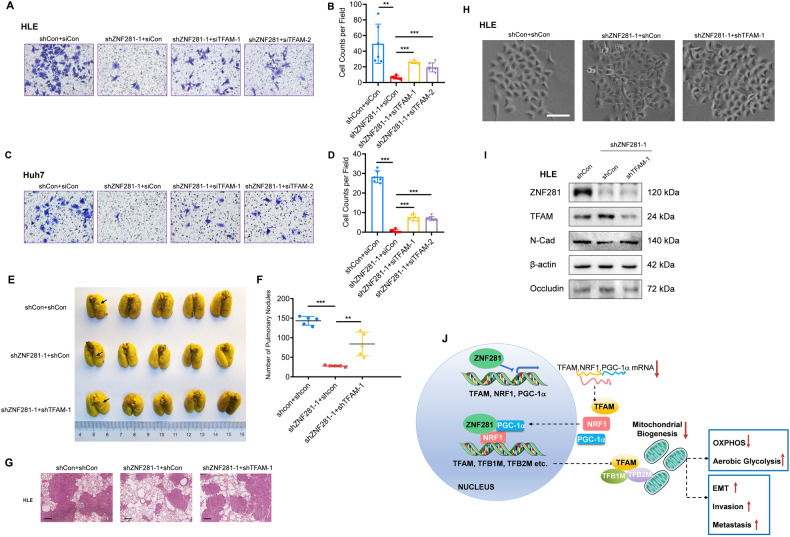
Increment of mitochondrial biogenesis suppresses ZNF281-mediated EMT, invasion and metastasis of HCC. **A, C** Matrigel transwell assays (10×) for cancer cell invasion in ZNF281 and TFAM knockdown HLE and Huh7 cells. **B, D** Quantification (*n* = 3; **p < 0.01, and ***p < 0.001) from (**A**) and (**C**). **E** Resected lungs for the groups as indicated. Arrows indicate pulmonary metastatic nodules with macroscopic observation. **F** Numbers of macroscopic visible pulmonary metastatic nodules. **G** Representative images of haematoxylin and eosin (H&E) staining for the resected lungs. Scale bars: 500 μm. **H** Representative phase contrast pictures of HLE clones transfected with shRNAs as indicated. Bar 100 μM. **I** Western blot showing N-cad and Occludin expression levels in ZNF281 and/or TFAM knockdown HLE cells. **J** Diagram of the role of ZNF281 in the regulation of mitochondrial biogenesis and function in hepatocellular carcinoma.

We wonder if inhibition of mitochondrial biogenesis contributes to metastasis promoting function of ZNF281. Simultaneous knockdown of TFAM significantly attenuated ZNF281 depletion-induced retardation of invasion of HCC cells, demonstrated with matrigel cell invasion assays (Fig. 8A–D). Meanwhile, the relatively weak effect on colony formation of ZNF281 depletion was reversed by TFAM shRNAs (Supplementary Fig. 6A, B). More importantly, ZNF281 knockdown resulted in pronounced reduction of lung tumor nodules in the pulmonary metastasis model with HLE cells in mice, which was largely reversed by co-knockdown of TFAM (Fig. 8E–G). Furthermore, ZNF281 depletion changed the HLE from mesenchymal morphology with spindle-like shape, protrusion formation, and scattered growth, to epithelial morphology with cuboid shape and obvious cell-cell adhesion, which was also dramatically attenuated by TFAM silencing (Fig. 8H). Meanwhile, the decrement of mesenchymal marker N-Cadherin, and the increment of epithelial marker Occludin upon ZNF281 knockdown, was also alleviated by TFAM shRNA (Fig. 8I). These results indicated that TFAM mediated mitochondrial biogenesis contributed to ZNF281-mediated EMT, invasion and metastasis in HCC.

## Discussion

In the present study, ZNF281 negatively regulates mitochondrial biogenesis and function through targeting the NRF1/PGC-1α-TFAM axis in HCC. With functional relevance, inhibition of mitochondrial biogenesis contributed to the pro-metastasis role of ZNF281.

ZNF281 regulates mitochondrial biogenesis through two mechanisms. First, ZNF281 directly bound onto the promoter regions of PGC-1α, NRF1, and TFAM and inhibited their expression. Second, ZNF281 interacted with NRF1 and PGC-1α to block their transcriptional regulation on TFAM, TFB1M, and TFB2M. ZNF281 thus added “double-brakes” to the expression of mitochondrial transcription factors, which make the regulation of ZNF281 on mitochondrial biogenesis more delicate. Together with other regulatory factors, mitochondrial biogenesis can be finely tuned in HCC cells.

ZNF281 promoted or repressed the expression of target genes by recruiting other cofactors. It recruits polycomb repressive complex 2 (PRC2) for transcriptional repression of GATA4, GATA6, and SOX17 to restrict ESC-to-XEN differentiation [23] while recruits NuRD complex to regulate Nanog autorepression somatic during cell reprogramming and cardiac reprogramming [7, 24]. Sin3A was also reported to interact with ZNF281 to restrict COMPASS-like H3K4 methyltransferase Mll2 in ESCs [25]. In HCC cells, several of its partners above promoted HCC progression. The PRC2 complex represses expression of AXIN2, activate Wnt/β-catenin pathway, and promote tumor development [26]. NuRD complex interacts with SALL4 to regulate stemness of EpCAM-positive hepatocellular carcinoma [27]. What cofactors did ZNF281 recruit to repress the expression of PGC-1α, NRF1 and their downstream targets remains unknown and needs further clarification.

PGC-1α functions as tumor suppressor in HCC [22], Our results showed direct repression of ZNF281 on PGC-1α, which partly explained the down-regulation of PGC-1α in HCC, and also suggested that silencing of PGC-1α might be an important mechanism for ZNF281 to promote invasion and metastasis of HCC. NRF1 regulates expression of multiple targets [28–32] to coordinate mitochondrial biogenesis and oxidative phosphorylation. We only detected the co-regulation of ZNF281/NRF1/PGC-1α on TFAM, TFB1M and TFB2M, whether other targets of NRF1/PGC-1α were also subjected to regulation by ZNF281 are currently unknown. GSEA analyses (Fig Supplementary Fig. 1C and D) indicated negative regulation of ZNF281 on oxidative phosphorylation. Meanwhile, many mitochondrial genes negatively correlate with of ZNF281 at the mRNA level. All these results strongly suggest regulation of ZNF281 on other mitochondrial expressed genes.

ZNF281 was firstly identified as an EMT driver in cancer, which prompted us to explore the functional relevance between mitochondrial biogenesis and EMT, invasion, and metastasis, in HCC. Knockdown of TFAM significantly attenuated the impaired invasion and metastasis of HCC cells induced by ZNF281 depletion (Fig. 8A–D), indicating inhibition of mitochondrial biogenesis contributed to ZNF281-mediated HCC invasion and metastasis. Several mechanisms may underlie this phenomenon: First, mtDNA content alteration may be involved in this attenuation, as reducing mtDNA with EtBr treatment or TFAM knockdown can activate calcineurin-mediated mitochondrial retrograde signals, and up-regulates the IGF-Akt and TGF-β pathways, and promotes EMT [33]; Second, reprogrammed metabolism accompanying mitochondrial biogenesis may drive ZNF281-mediated EMT and metastasis in HCC. A recent study indicated that TFAM loss blocked the TCA cycle, increased the intracellular malonyl-CoA and malonylation of mDia2, which in turn drives actin assembly and pulmonary metastasis in HCC [34], further supporting our results.

In summary, this study clarified the important roles and molecular mechanisms of ZNF281 in regulating mitochondrial biogenesis in HCC, which facilitates tumor invasion and metastasis. Our study may benefit the understanding of the pathophysiology of HCC, and help the development of novel therapies for this disease.

## Materials and methods

### Cell culture

The human liver cancer cell lines of Huh7 was purchased from the Cell Resource Center, Peking Union Medical College. The human liver cancer cell line HLE was kindly given by Dr. Dexi Chen at Beijing Institute of Hepatology. Huh7 and HLE cells were maintained in Dulbecco’s modified Eagle medium (DMEM) and were supplemented with 10% fetal bovine serum (FBS, Gibco, New York, NY, USA) and 1% Penicillin/Streptavidin (KeyGene, MD, USA) in Corning (New York, NY, USA) dishes at 37 °C in a humidified incubator with 5% CO_2_. The identity of the cell lines was confirmed with its suppliers, continuing culture in suggested conditions, and observation under microscopy for cell morphology as well as growth rate.

### SiRNA transfection

SiRNAs targeting ZNF281 were purchased from Beijing Syngen Tech Co. Ltd. (Beijing, China). The sequence of the oligos used were: siZNF281-1: 5′-GCACAUGUGGUGAAGUCAUTT-3′ (forward) and 5′-AUGACUUCACCACAUGUGCTT-3′ (reverse); siZNF281-2: 5′-CCAGAUAGAUCCUCAGAAATT-3′ (forward) and 5′-UUUCUGAGGAUCUAUCUGGTT-3′ (reverse). The siRNAs targeting TFAM were purchased from General Biosystems Co. Ltd. (Anhui, China). The sequences used were as, siTFAM-1: 5′-UAUCUCUUCUUUAUAUACCTT-3′ (forward) and 5′-GGUAUAUAAAGAAGAGAUATT-3′ (reverse); siTFAM-2: 5′-AUUUCAUUAUGAUAACGAGTT-3′ (forward) and 5′-CUCGUUAUCAUAAUGAAAUTT-3′ (reverse). The siRNA targeting NRF1 were purchased from General Biosystems Co. Ltd. (Anhui, China). The sequences used were as, siNRF1: 5′-GGGCAAAUGUCCGGAGUGATT-3′ (forward) and 5′-UCACUCCGGACAUUUGCCCTT-3′ (reverse). A scrambled siRNA was used as the common negative control for all siRNA experiments. Cells were transfected with siRNAs (50 nM in final concentration) using Lipofectamine RNAIMAX (Invitrogen, Carlsbad, CA, USA) following the manufacturer’s instructions.

### Lentivirus infection and preparation of stable transgenic cells

Cells were seeded in 6-well plate at a density of 2 × 10^5^ per well and cultured for 24 h. For knockdown of ZNF281, the lentivirus pHS-ASR-LW007 (Shcon), pHS-ASR-LW125 (ShZNF281-1, with the same target sequence as siZNF281-1 mentioned above) or pHS-ASR-LW126 (ShZNF281-2, with the same target sequence as siZNF281-1 mentioned above) purchased from Beijing Syngen Tech Co.Ltd. (Beijing, China) were added at the multiplicity of infection (MOI) of 10 for 12 h; for concomitant knockdown of ZNF281 and TFAM, the lentivirus pHS-ASR-LW007 (Shcon), pHS-ASR-LW125 (ShZNF281-1) and the lentivirus HBLV-mcherry-BSD NC (Shcon) or HBLV-h-TFAM shRNA1-mCherry-BSD (ShTFAM) purchased from Hanbio Tech Co. Ltd. (Shanghai, China) were added in combination at the multiplicity of infection (MOI) of 10 for 12 h. The transduction efficiency was assessed using a fluorescence microscopy at 96 h post infection. After the selection in 2 μg/ml puromycin (for ShZNF281) or 5 ng/ml blasticidin (ShTFAM) for 7 days, the obtained cells were verified by western blotting.

### RNA extraction and quantification

Total RNAs from treated cells were extracted using RNeasy Mini Kit (Yishan, Shanghai, China) according to the manufacturer’s suggestions. One microgram of total RNA was subjected to reverse transcription using the Reverse Transcription Kit (Takara, Dalian, China). The RT-qPCR primers for the genes detected were shown in Table 3. Real-time PCR assays were then performed with SYBR Green (Novoprotein, Shanghai, China) in a Rocgene Archimed X6 Real-Time PCR Detection System. 18 S were used as an internal control. The fold changes of respective genes were calculated with the 2^-ΔΔCt^ method.

**Table 3:** Primers used for RT-qPCR reaction.

Gene	Forward	Reverse
ZNF281	TCTTCACCTCTCCACAACCAC	TGTAGCATCCAAAGCAGACAA
18 S	GTAACCCGTTGAACCCCATT	CCATCCAATC GGTAGTAGCG
TFAM	CCAAAAAGACCTCGTTCAGCTT	CTTCAGCTTTTCCTGCGGTG
PGC-1α	GCTTTCTGGGTGGACTCAAGT	GAGGGCAATCCGTCTTCATCC
NRF1	CTTATCCAGGTTGGTACGGGG	CTCACCTCCCTGTAACGTGG
TFB1M	GTCCTGGGTGAGGTAGGGT	TTTCCTTACAATCTTATCTGTCAGC
TFB2M	GGAGTGCAATCCAGGTCC	GAGCCCTCGAGAAGACATAGC
MRPL12	CTCAACGAGCTCCTGAAGAAAA	ACGGTGAAATGTGTCCGTTCT
NDUFS3	GTTCTGTTGCTGCCGGTGA	CAGGACACCTGAACTTGTTGGA
SDHB	TATGCAGGCCTATCGCTGGA	ATGGTGTGGCAGCGGTATAG
BCS1L	GTCACGGCGGTTTTCGTAAC	TCCAGCCCCAAAGTAGGGAT
COX6B1	CGGGGTGCCTTTAGGATTCA	TTCTGACAGCGGTGGAAGTC
ATPAF2	GGAGGAGGAGTACCAGATCCA	GCTTGTGCTTGACTGTGGTG
MT-CO2	CCTGCGACTCCTTGACGTTG	AGCGGTGAAAGTGGTTTGGTT

### Western blot and co-immunoprecipitation (Co-IP)

Total proteins were extracted with RIPA cell lysis buffer containing the cocktail of protease inhibitors, and protein concentration was quantified with a BCA Protein Assay Kit (Novagen, Billerica, MA, USA). A loading of 12 μg total protein each sample was subjected to separation in a 10% SDS–PAGE gel, and then electro-transferred to polyvinylidene fluoride (PVDF) filters (Merck Millipore, Darmstadt, Germany). The filters were then blocked for 1 h with 1.5% BSA in TBST (pH 8.0) and incubated with the corresponding primary antibodies overnight at 4 °C. Primary antibodies used includes anti-ZNF281 (1:1000, Abcam, Cambridge, MA, USA), anti-TFAM (1:1000, Cell Signaling Technology Inc., Danvers, MA, USA), anti-NRF1 (1:1000, Cell Signaling Technology Inc., Danvers, MA, USA), anti-PGC-1α (1:1000, Millipore, Billerica, MA, USA), anti-N-cadherin (1:1000, Cell Signaling Technology Inc., Danvers, MA, USA), anti-Occludin (1:1000, Cell Signaling Technology Inc., Danvers, MA, USA) and β-actin (1:10000, ABclonal, Wuhan, China). Following incubation with HRP conjugated goat anti-mouse/rabbit IgG secondary antibodies (SAB, MD, USA) at room temperature for 1 h, the bands were imaged using a MiniChemi™ Chemiluminescence instrument. In co-immunoprecipitation assays, proteins were extracted from HLE cells using RIPA buffer (50 mM Tris-Cl (pH 7.5), 150 mM NaCl, 0.2 mM EDTA and 2% NP-40) and incubated with primary anti-ZNF281 (1 μg, Abcam, Cambridge, MA, USA) or control IgG (1 μg, Applygen, Beijing, China), followed with immunoprecipitation using Protein A/G PLUS-Agarose (Santa Cruz, CA, USA) at 4 °C overnight. Collected samples were extensively washed with RIPA buffer prior to immunoblotting procedures.

### Immunohistochemistry for ZNF281 and ATP5B in tissue section array samples

A tissue microarray including 71 HCC tumor and paired para-tumor sections was constructed from patients underwent surgical resection at You’ An hospital, and informed consent documents were obtained. The study concerning tissue array was performed in compliance with the Helsinki Declaration, and approved by the Ethic Committee of You’An hospital affiliated to Capital Medical University (#LL-2021-163-K). The tissue microarray was incubated with anti-ZFP281 (1:200, Abcam, Cambridge, MA, USA) and anti-ATP5B (1:200, Santa Cruz, CA, USA) as the primary antibody, respectively. The stained IHC samples were examined under a microscope and scored from 0 to 12 based on ZNF281 staining intensity. The average scores from the evaluation of 4 independent investigators were used for statistical analyses.

### In vivo pulmonary metastasis assay

The experimental procedures were conducted in accordance with the Guidelines for Welfare of Animals in Experimental Neoplasia, and the protocols were approved by the Institutional Animal Care and Use Committee of Capital Medical University. All animal experiments were reported in accordance with ARRIVE guidelines. For lung metastasis assay, fifteen male 5-week-old BALB/c nude mice were randomized into three groups (*n* = 5 per group), HLE cells (1 × 10^6^) stably transfected with shRNAs against ZNF281 together with or without shRNAs for TFAM were injected into the tail veins to establish pulmonary metastasis model. Six weeks after the injection, mice were sacrificed, the lungs were removed, stained with 5% picric acid. The pulmonary metastatic foci were detected through hematoxylin and eosin (H&E) staining. The animal experiments were performed with single blind design.

### Matrigel transwell assay

Costar 24-well transwell plates of 8 μm in pore size were used for matrigel transwell assays. The filters were coated with matrigel (BD Biosciences, CA, USA) at a concentration of 1:30 dilution in DMEM without FBS. Then 2 × 10^4^ cells were seeded onto the matrigel in the upper chamber. The lower chamber was filled with DMEM containing 20% FBS. The plates were maintained in cultures for 24 h, then the cells remained in the upper chamber on the filter were removed. Cells invading to the lower chamber were fixed with 1% paraformaldehyde for 30 min and then stained with 0.5% Crystal Violet Dyes for 15 min. The air-dried filters were imaged under a microscope. Six different fields per condition were counted.

### Immunofluorescence

HLE and Huh7 cells following different treatments were seeded in confocal dishes (NEST, VA, USA) and maintained in DMEM for 24 h. The cells were fixed with 4% paraformaldehyde and permeabilized with 0.1% Triton X-100, and then incubated with primary antibodies overnight at 4 ˚C. Antibodies were used as follows: anti-ZNF281 (1:50, Santa Cruz, CA, USA), anti-TFAM (1:50, Cell Signaling Technology Inc., Danvers, MA, USA), anti-NRF1 (1:50, Cell Signaling Technology Inc., Danvers, MA, USA), anti-PGC-1α (1:50, Millipore, Billerica, MA, USA), anti-SDHB (1:200, Abcam, Cambridge, MA, USA), and anti-MT-CO2 (1:200, Abcam, Cambridge, MA, USA). The fluorescent secondary antibodies used were Alexa fluor (m) 594 goat A or Alexa fluor (R) 488 goat A (1:200, Invitrogen, NY, USA). Counter-staining of the nuclei was performed using 4′, 6-diamidino-2-phenylindole (DAPI). Samples were observed under a Leica DM5000 B fluorescent microscope and Leica SP5 confocal microscope (Leica Microsystems, Wetzlar, Germany).

### Relative mtDNA content measurements

The level of mtDNA was measured by assessing the relative levels of mtDNA-ND1 to nDNA-B3M using a PCR analysis of total DNA extracted from human HLE and Huh7 cells. The following amplification primers (5′ to 3′) were described previously [35]: mtDNA-ND1 (sense, CCCTAAAACCCGCCACATCT; antisense, GAGCGATGGTGAGAGCTAAGGT) and nDNA-B3M (sense, TGCTGTCTCCATGTTTGATGTATCT; antisense: TCTCTGCTCCCCACCTCTAAGT).

### MitoTracker and MitoSOX staining

Cells were incubated with 50 nM MitoTracker Green FM probe (Thermo Fisher Scientific, M7514) or 5 μM MitoSOX reagent (Thermo Fisher Scientific, M36008) at 37 °C for 30 min. Cells were then washed and analyzed by flow cytometry (Excitation wavelength 488 nm and 594 nm).

### Colony formation assays

For colony formation assay, 500 cells were seeded in a 6-cm cell culture dish and cultured for 14 days. Then the culture medium was removed, and the colonies were fixed with 4% paraformaldehyde (PFA) and stained with 0.5% crystal violet.

### Lactic acid release

For lactic acid release analyses, the HCC cells were seeded in 12-well plates at a density of 1.5 × 10^5^ cells per well, and control wells containing only medium. After 24 h, the culture medium was collected using a pipette and the total volume for each well was recorded, which was then placed in a glucose lactate glutamate analyzer (Shenzhen Sieman Technology Co., Ltd.), and the release of lactic acid was detected.

### Oxygen consumption rate (OCR) measurement in cells

The mitochondrial respiratory capacity was determined using XF Cell Mito Stress Test Kit (Agilent Technologies, 103015–100). Cells were seeded in the XF96 cell culture microplate at a density of 2 × 10^4^ cells per well. Microplates was incubated for 24 h at 37 °C. Seahorse XF96 FluxPak sensor cartridge was hydrated in the utility plate and filled with 200 ml of Seahorse Calibrant overnight in a non-CO_2_ incubator at 37 °C. Then, the cells were incubated with the base medium containing 2 mM L-glutamine, 1 mM sodium pyruvate, and 10 mM glucose for 1 h prior to assay. The oxygen consumption rate (OCR) was measured by XFe96 extracellular flux analyzer with sequential injection of 1 mM oligomycin A, 1 mM FCCP, and 0.5 mM rotenone/antimycin A. Finally, the cell number was counted and the OCR value was normalized by the number of cells per well.

### Chromatin immunoprecipitation (ChIP) assay

A ChIP Assay Kit (Cell Signaling Technology Inc., Danvers, MA, USA) was used following the manufacturer’s instructions. The DNA-bound protein was immunoprecipitated using anti-ZFP281 (Abcam, Cambridge, MA, USA) antibody and NRF1 (Cell Signaling Technology Inc., Danvers, MA, USA) antibody. To quantify co-precipitated DNAs, samples were subjected to PCR amplification, employing primers in Table 4. The enrichment ratio was determined according to the following formula: (% IP/INPUT = 2[(Ct (x% input) – log (x %) /log2) – Ct (IP)] × 100).

**Table 4:** Primers used for ChIP-qPCR.

Gene	Forward	Reverse
NRF1 (Predicted ZNF281 binding)	AAACCGCCACCCCGAGGAT	TCGCCCAAAGAGCCAGCCT
PGC-1α (Predicted ZNF281 binding)	TGTAGTAAGACAGGTGCCTTCAG	GCTCAAGCCATCTCAGAGCC
TFAM (Predicted ZNF281 binding)	GGACAGAGGTGGCTCAACAGCG	CCAGACCTTCCCAGGGCACTCA
TFB1M (Known NRF1 binding)	CCTAGTCCACCCGGCTCT	GAGGAACCTGCGAGACCTAA
TFB2M (Known NRF1 binding)	ACGGTCCACTCACAATCCTC	CCCACGTGGAACATTTTCTG
TFAM (Known NRF1 binding)	TCTACCGACCGGATGTTAGC	CTTCCCAGGGCACTCAGC

### Targeted metabolomics

For targeted metabolomics, aspirate the cell medium completely and gently wash the cells using PBS 3 times, then put the plates on dry ice and add 2 ml of 80% (vol/vol) methanol (pre-chilled to −80 °C) and incubate the plates at −80 °C for 2 h. Scrape the plates on dry ice with cell scraper. Transfer the cell lysate/methanol mixture to a 1.5-ml tube on dry ice. Centrifuge the tube at 14,000 g for 20 min at 4 °C and transfer the metabolite-containing supernatant to a new 1.5-ml tube on dry ice. Lyophilize the supernatants with speedvac at room temperature. Analysis was performed on TSQ Quantiva Triple Quadrupole mass spectrometer (Thermo, CA) with positive/negative ion switching at the Metabolomics Facility at Tsinghua University Branch of China National Center for Protein Sciences (Beijing, China).

### Statistical analyses

Data were presented as mean ± SEM (standard error of mean). Statistical analyses were performed using GraphPad Prism 8.0. Shapiro-Wilk normality test was first employed before analyses to assess data distribution. For data of normal distribution, unpaired two tailed t-tests test was used for comparison of two groups while one-way ANOVA followed by Tukey’s multiple comparisons were performed for multiple groups. The data in immunohistochemistry in tissue array did not meet normal distribution or uneven variance, and the nonparametric Mann-Whitney U test was used. *P* < 0.05 was considered to be statistically significant.

### Supplementary information

Supplementary figures and legends

Full and uncropped western blots
