# Supplementary material for: ZNF281 inhibits mitochondrial biogenesis to facilitate metastasis of hepatocellular carcinoma
Source: Cell Death Discov. 2023 Oct 25;9:396. doi: 10.1038/s41420-023-01691-9 (PMC10600106; doi:10.1038/s41420-023-01691-9)
Supplement: Supplementary file 1 — Supplementary figures and legends [file 41420_2023_1691_MOESM1_ESM.docx]

**Supplementary figures**

**
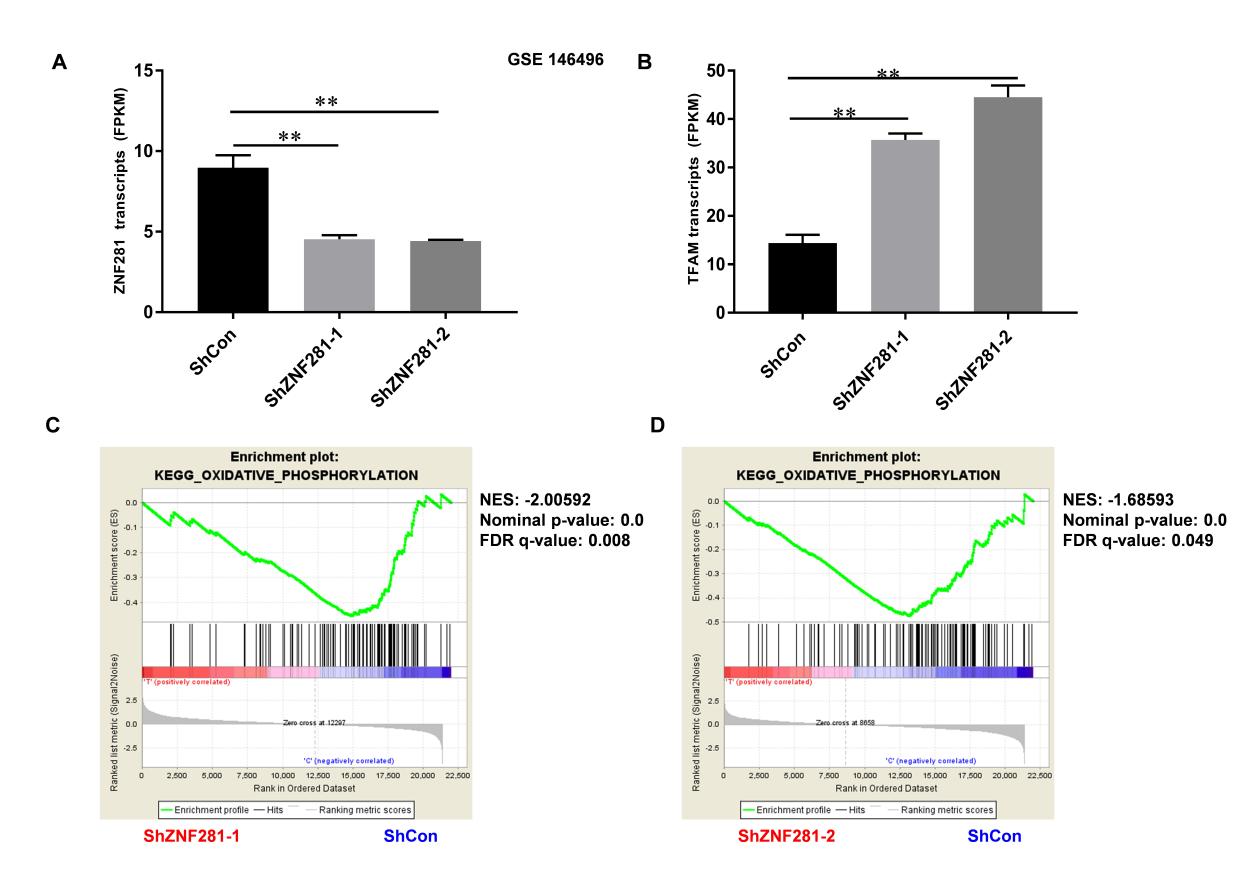
**

**Supplementary Fig. 1.** **ZNF281 negatively correlates with TFAM expression and oxidative phosphorylation in HCC cells, related to figure 1.**

**A-B** HLE cells were infected with two different ZNF281 shRNAs in lentiviruses vectors, and the mRNA FPKM of ZNF281 and TFAM were detected by RNA-Seq (n=3; ***p*<0.01).

**C-D** GSEA showed negative enrichment of oxidative phosphorylation pathway with Shcon HLE cells (n=3; **p*<0.05).


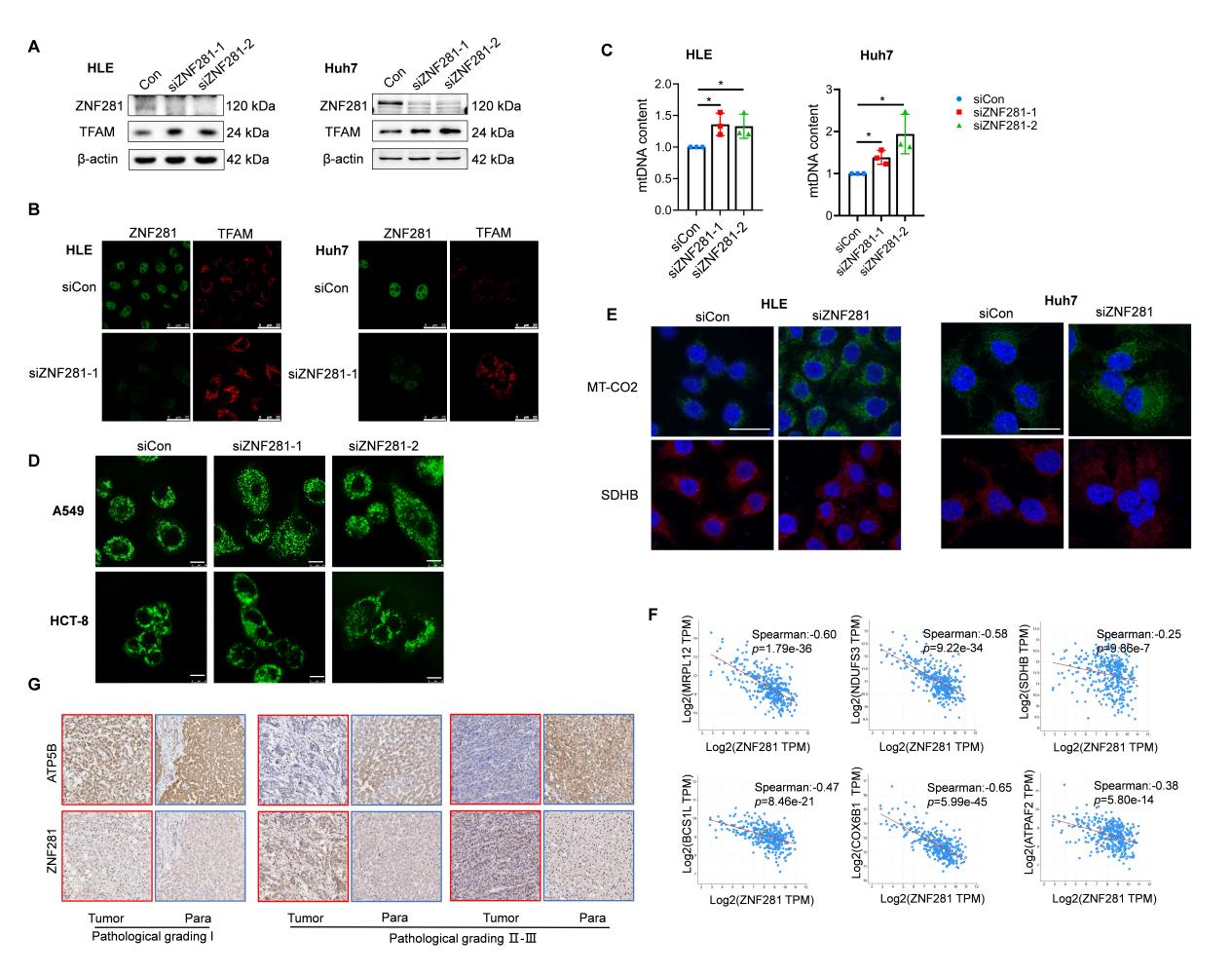


**Supplementary Fig. 2. ZNF281 negatively regulates mitochondrial biogenesis in cancer cells, related to figure 1.**

**A** Western blotting for TFAM following the infection with siCon and siZNF281 in HLE and Huh7 cells.

**B** Cellular immunofluorescence was used to detect the expression and subcellular localization of TFAM in ZNF281 Knockdown HLE and Huh7 cells. Scale bars, 10 μm.

**C** Relative mtDNA content was measured by qPCR in ZNF281 transient knockdown HLE and Huh7 cells (n=3; **p*<0.05).

**D** Confocal laser scanning microscope analysis of mitochondrial mass in A549 and HCT-8 cancer cells after mitotracker green staining. Scale bars, 25 μm.

**E** Immunofluorescence of SDHB and MT-CO2 in HLE and Huh7 cells with ZNF281 knockdown. Scale bars, 25 μm.

**F** Correlation analyses between ZNF281 and mitochondrial expressed genes at the mRNA level from the TCGA liver cancer database.

**G** Representative immunohistochemical staining images of ZNF281 and ATP5B from HCC tissues. Consecutive tissue sections were used for same patient.

**
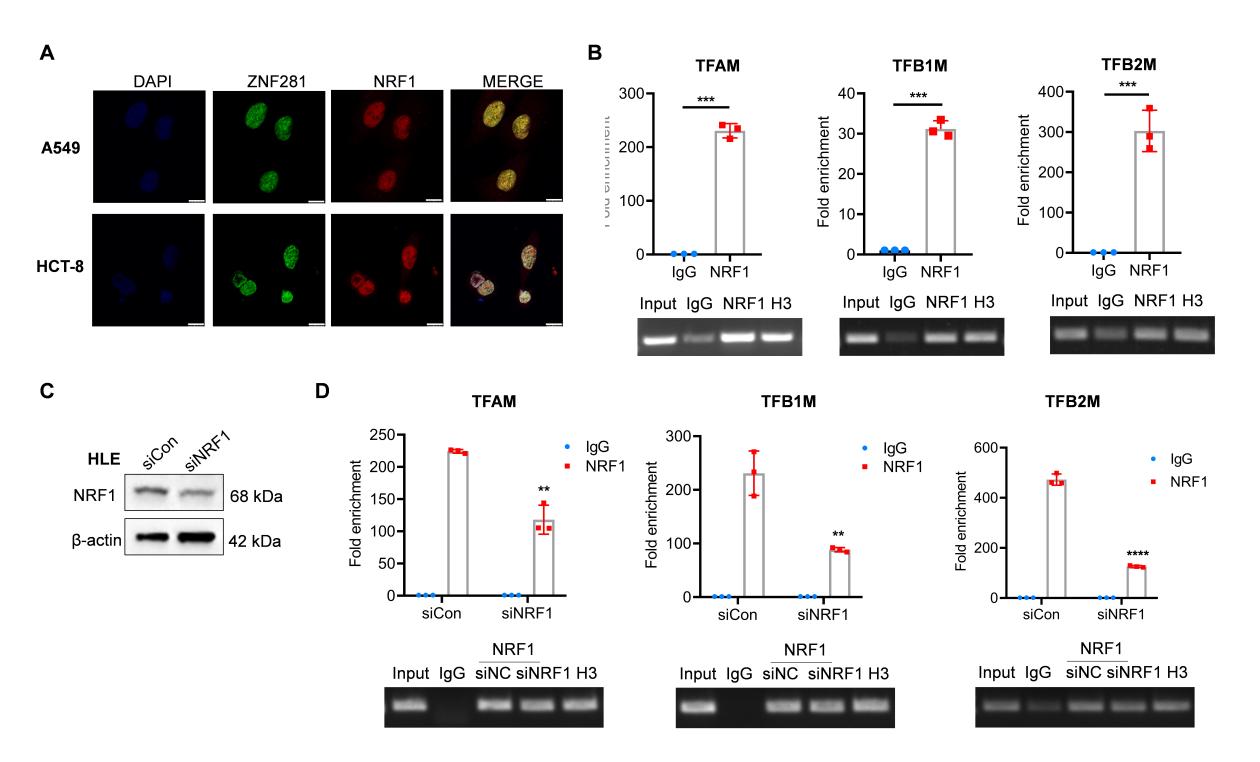
**

**Supplementary Fig. 3. NRF1 regulates TFAM, TFB1M and TFB2M, related to figure 3.**

**A** Immunofluorescence assay to detect the co-localization of ZNF281 and NRF1 in A549 and HCT-8 cells (Scale bar: 10 μm).

**B** Upper: ChIP-qPCR demonstrating the enrichment of NRF1 in the promoter regions of TFAM/TFB1M/TFB2M. Lower: analysis of ChIP-qPCR results by semi-quantitative PCR and agarose gel electrophoresis (n=3; ****p*<0.001).

**C** Western blotting verified the knockdown efficiency of NRF1.

**D** Upper: ChIP-qPCR demonstrating the enrichment of NRF1 in the promoter regions of TFAM/TFB1M/TFB2M in NRF1 knockdown HLE cells. Lower: analyses of ChIP- results by semi-quantitative PCR and agarose gel electrophoresis (n=3; ***p*<0.01, and ****p*<0.001).


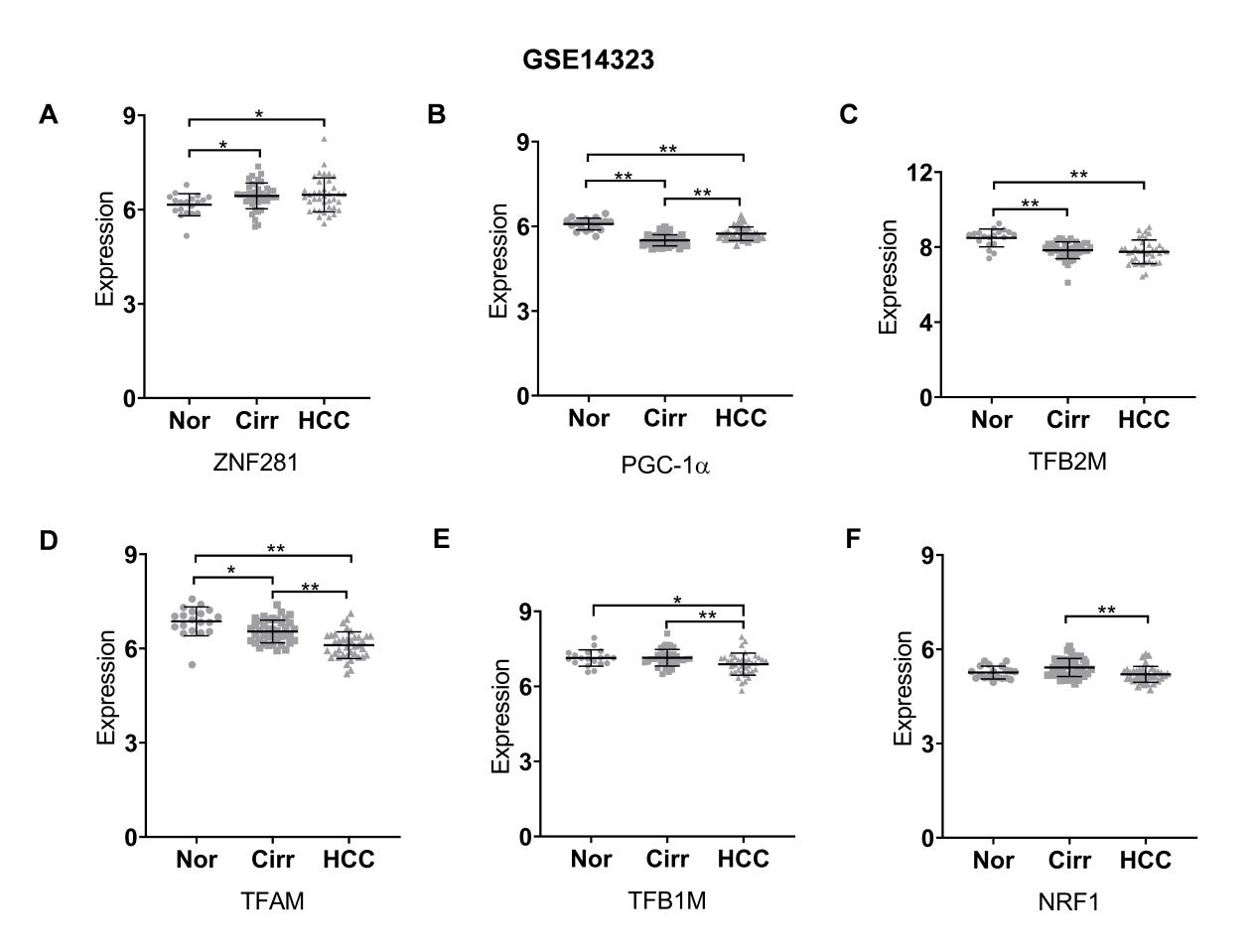


**Supplementary Fig. 4. Expression of ZNF281 was up-regulated while PGC-1α/NRF1/TFAM was down-regulated.**

1. **F** The expression of ZNF281, PGC-1α, NRF1, TFAM, TFB1M and TFB2M in normal tissues, liver cirrhosis, and liver cancer tissues from GSE14323 (n=3; **p*<0.05, ***p*<0.01, and ****p*<0.001).


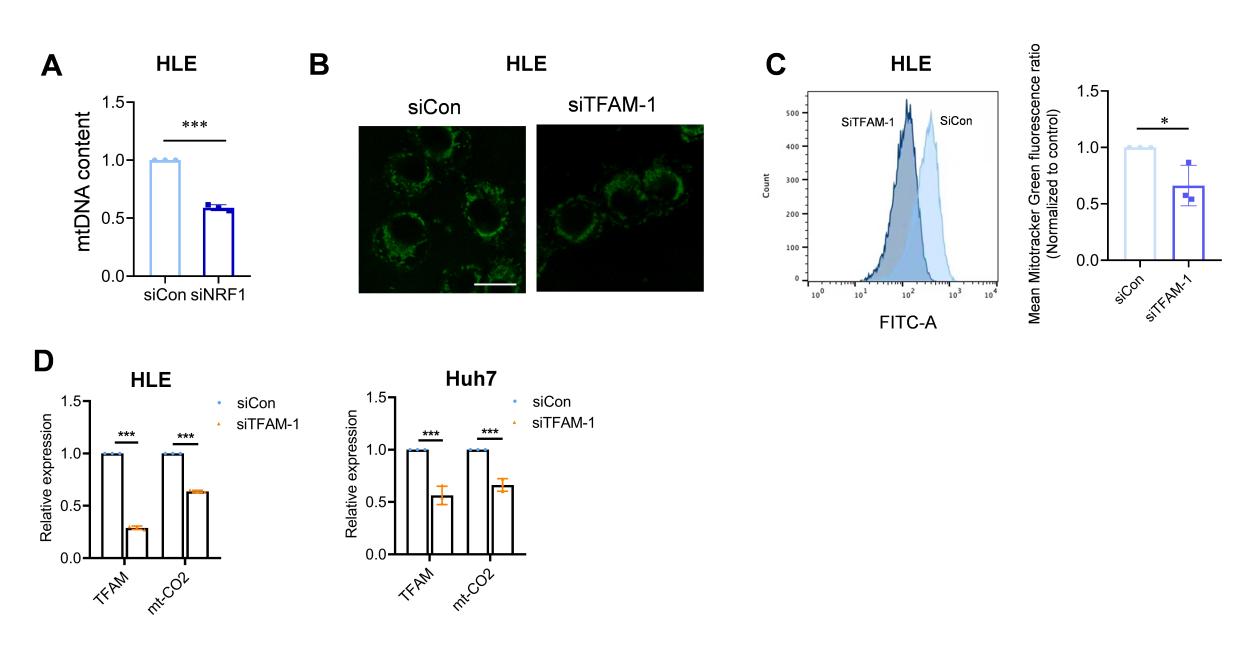


**Supplementary Fig. 5. TFAM or NRF1 positively regulates mitochondrial biogenesis.** Related to figure 6.

**A** Relative mtDNA content was measured by qPCR in NRF1 transient knockdown HLE cells (n=3; ****p*<0.001).

**B** Confocal laser scanning microscope analysis of mitochondrial mass in TFAM transient knockdown HLE cells with mitotracker green staining. Scale bars, 25 μm.

**C** Left: Flow cytometry analyses of mitochondrial mass alteration with mitotracker green staining in HLE cells; Right: statistical analyses of fluorescence (n=3; *p<0.05).

**D** mRNA levels of MT-CO2 was measured by RT-qPCR in TFAM knockdown HLE and Huh7 cells (n=3; ****p*<0.001) .


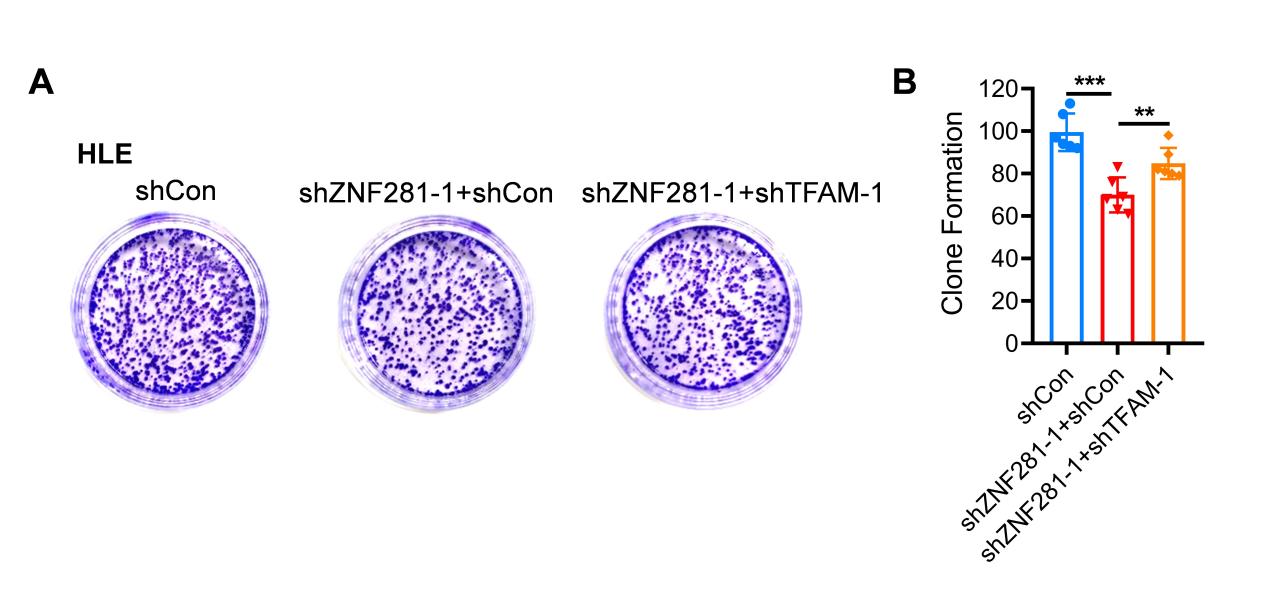


**Supplementary Fig. 6. Increment of mitochondrial biogenesis suppresses ZNF281-mediated growth of HCC cells, related to figure 7.**

**A** Colony formation verified the effects of ZNF281 and/or TFAM knockdown on cell growth.

**B** Randomly 6 fields of view on each petri dish in (A), and statistical analysis on the number of cell clones formed was performed (n=3; ***p*<0.01, and ****p*<0.001).

### Supplementary Table S1 TCGA database analysis of the correlation between the mRNA expression levels of ZNF281 and mitochondrial related genes in HCC [(TCGA, Firehose Legacy)](https://www.cbioportal.org/study?id=lihc_tcga)

| Mitochondrial related genes | Correlation with ZNF281  (Spearman’s correlation coefficient) | *p*-Value |
| --- | --- | --- |
| NDUFS3 | -0.580 | 9.22e-34 |
| NDUFS4 | -0.342 | 2.46e-11 |
| NDUFS5 | -0.593 | 1.31e-35 |
| NDUFS6 | -0.595 | 7.17e-36 |
| NDUFS7 | -0.513 | 1.48e-25 |
| NDUFS8 | -0.638 | 1.36e-42 |
| NDUFV1 | -0.406 | 1.00e-15 |
| NDUFV2 | -0.459 | 3.44e-20 |
| NDUFV3 | -0.429 | 1.48e-17 |
| NDUFA12 | -0.554 | 2.71e-30 |
| NDUFA13 | -0.663 | 7.50e-47 |
| NDUFA4 | -0.438 | 2.85e-18 |
| NDUFAF4 | -0.472 | 2.35e-21 |
| NDUFA11 | -0.655 | 1.71e-45 |
| NDUFAF1 | -0.128 | 0.0153 |
| NDUFAB1 | -0.416 | 1.81e-16 |
| NDUFAF8 | -0.623 | 3.88e-40 |
| NUDFA3 | -0.619 | 1.77e-39 |
| NDUFA2 | -0.619 | 2.09e-39 |
| NDUFAF3 | -0.615 | 8.47e-39 |
| NDUFA7 | -0.576 | 3.81e-33 |
| NDUFAF2 | -0.574 | 6.91e-33 |
| NDUFA8 | -0.555 | 1.74e-30 |
| NDUFA6 | -0.539 | 1.47e-28 |
| NDUFA1 | -0.507 | 7.35e-25 |
| SDHA | -0.0500 | 0.344 |
| SDHB | -0.255 | 9.86e-7 |
| SDHAF1 | -0.556 | 1.17e-30 |
| SDHAF2 | -0.622 | 5.49e-40 |
| BCS1L | -0.466 | 8.46e-21 |
| UQCRB | -0.578 | 1.73e-33 |
| UQCRQ | -0.522 | 1.42e-26 |
| UQCRC1 | -0.456 | 7.18e-20 |
| UQCRH | -0.449 | 2.98e-19 |
| UQCRFS1 | -0.323 | 3.32e-10 |
| UQCR10 | -0.630 | 3.76e-41 |
| UQCR11 | -0.589 | 5.05e-35 |
| UQCC3 | -0.670 | 3.70e-48 |
| UQCRHL | -0.361 | 1.54e-12 |
| UQCC2 | -0.575 | 4.81e-33 |
| SURF1 | -0.474 | 1.39e-21 |
| SCO1 | -0.0412 | 0.436 |
| SCO2 | -0.541 | 1.05e-28 |
| COX6B1 | -0.652 | 5.99e-45 |
| COX4I1 | -0.559 | 5.35e-31 |
| COX6A1 | -0.593 | 1.46e-35 |
| COX6C | -0.605 | 2.41e-37 |
| COX7A2 | -0.506 | 9.02e-25 |
| COX7A2L | -0.220 | 2.453e-5 |
| COX7B | -0.482 | 2.56e-22 |
| COX7C | -0.555 | 1.76e-30 |
| COX5A | -0.506 | 7.95e-25 |
| COX5B | -0.553 | 3.05e-30 |
| COX8A | -0.527 | 4.28e-27 |
| COX14 | -0.592 | 1.93e-35 |
| COX17 | -0.590 | 3.84e-35 |
| COX16 | -0.495 | 1.07e-23 |
| COX4I2 | -0.369 | 5.02e-13 |
| COX7A1 | -0.366 | 7.36e-13 |
| COX6B2 | -0.183 | 4.959e-4 |
| COX7A2L | -0.220 | 2.453e-5 |
| ATPAF2 | -0.382 | 5.80e-14 |
| ATP5F1E | -0.688 | 9.27e-52 |
| ATP5MPL | -0.640 | 8.08e-43 |
| ATP5MD | -0.609 | 6.30e-38 |
| ATP5ME | -0.608 | 9.85e-38 |
| ATP5MC2 | -0.606 | 1.82e-37 |
| ATP5F1EP2 | -0.595 | 6.59e-36 |
| ATP5MF | -0.594 | 9.46e-36 |
| ATP5F1D | -0.581 | 7.33e-34 |
| ATP5PD | -0.562 | 2.58e-31 |
| ATP5MG | -0.555 | 1.74e-30 |
| ATP5F1B | -0.119 | 0.0237 |
| MRPL12 | -0.599 | 1.79e-36 |
| MRPL52 | -0.737 | 7.78e-63 |
| MRPL27 | -0.655 | 1.55e-45 |
| MRPL23 | -0.642 | 3.53e-43 |
| MRPL47 | -0.633 | 9.44e-42 |
| MRPL17 | -0.623 | 3.93e-40 |
| MRPS15 | -0.620 | 1.17e-39 |
| MRPL14 | -0.607 | 1.47e-37 |
| MRPS11 | -0.581 | 7.74e-34 |
| MRPS26 | -0.574 | 6.52e-33 |
| MRPS48 | -0.580 | 8.81e-34 |
| MRPS24 | -0.566 | 7.29e-32 |
| MRPL53 | -0.586 | 1.31e-34 |
| MRPL28 | -0.606 | 2.05e-37 |
| MRPL51 | -0.600 | 1.40e-36 |
| MRPL21 | -0.595 | 7.90e-36 |
| MRPS26 | -0.574 | 6.25e-33 |
| MRPL38 | -0.569 | 3.09e-32 |
| MRPS12 | -0.568 | 3.46e-32 |
| MRPL22 | -0.550 | 6.83e-30 |
| TOMM40 | -0.543 | 4.85e-29 |
| TOMM6 | -0.535 | 4.96e-28 |
| TOMM7 | -0.512 | 1.95e-25 |
| TOMM5 | -0.510 | 2.95e-25 |
| TOMM22 | -0.486 | 9.65e-23 |
| TOMM34 | -0.311 | 1.62e-9 |
| TOMM20L | -0.179 | 6.288e-4 |
| TIMM22 | -0.330 | 1.31e-10 |
| TIMM23 | -0.551 | 5.25e-30 |
| TIMM13 | -0.634 | 7.18-42 |
| TIMM50 | -0.606 | 1.55e-37 |
| TIMM10 | -0.552 | 3.86e-30 |
| TIMM17B | -0.515 | 9.07e-26 |
| TIMM9 | -0.455 | 9.17e-20 |
| TIMM44 | -0.400 | 2.83e-15 |
| TIMM29 | -0.356 | 3.47e-12 |
| TIMMDC1 | -0.324 | 3.11e-10 |
| TIMM8A | -0.311 | 1.63e-9 |
| CYC1 | -0.506 | 7.55e-25 |
| SIRT3 | -0.453 | 1.16e-19 |
| SITR4 | -0.336 | 6.32e-11 |

### Supplementary Table S2 TCGA databases analyses of the correlation between the mRNA expression levels of ZNF281 and mitochondrial related genes in [Colorectal Adenocarcinoma (TCGA, PanCancer Atlas)](https://www.cbioportal.org/study?id=coadread_tcga_pan_can_atlas_2018)

| Mitochondrial related genes | Correlation with ZNF281  (Spearman’s correlation coefficient) | *p*-Value |
| --- | --- | --- |
| NDUFS3 | -0.517 | 4.09e-37 |
| NDUFS4 | -0.0971 | 0.0263 |
| NDUFS5 | -0.406 | 3.13e-22 |
| NDUFS6 | -0.448 | 3.12e-27 |
| NDUFS7 | -0.539 | 8.87e-41 |
| NDUFS8 | -0.554 | 1.98e-43 |
| NDUFV1 | -0.501 | 1.08e-34 |
| NDUFV3 | -0.229 | 1.23e-7 |
| NDUFA12 | -0.284 | 3.37e-11 |
| NDUFA13 | -0.581 | 1.48e-48 |
| NDUFA4 | -0.179 | 3.819e-5 |
| NDUFAF4 | -0.0573 | 0.190 |
| NDUFA11 | -0.522 | 5.84e-38 |
| NDUFAB1 | -0.246 | 1.08e-8 |
| NDUFAF8 | -0.531 | 2.15e-39 |
| NUDFA3 | -0.561 | 8.95e-45 |
| NDUFA2 | -0.476 | 6.17e-31 |
| NDUFAF3 | -0.462 | 4.00e-29 |
| NDUFA7 | -0.575 | 1.73e-47 |
| NDUFAF2 | -0.270 | 3.29e-10 |
| NDUFA8 | -0.426 | 1.63e-24 |
| NDUFA6 | -0.391 | 1.23e-20 |
| NDUFA1 | -0.348 | 2.38e-16 |
| SDHA | -0.291 | 1.03e-11 |
| SDHB | -0.186 | 1.861e-5 |
| SDHAF1 | -0.444 | 9.99e-27 |
| SDHAF2 | -0.313 | 2.19e-13 |
| BCS1L | -0.451 | 1.49e-27 |
| UQCRB | -0.139 | 1.383e-3 |
| UQCRQ | -0.496 | 7.72e-34 |
| UQCRC1 | -0.580 | 2.01e-48 |
| UQCRH | -0.271 | 2.95e-10 |
| UQCRFS1 | -0.184 | 2.352e-5 |
| UQCR10 | -0.562 | 5.90e-45 |
| UQCR11 | -0.540 | 4.65e-41 |
| UQCC3 | -0.575 | 2.07e-47 |
| UQCRHL | -0.289 | 1.49e-11 |
| UQCC2 | -0.503 | 5.55e-35 |
| SURF1 | -0.324 | 2.76e-14 |
| SCO2 | -0.406 | 3.12e-22 |
| COX6B1 | -0.589 | 2.80e-50 |
| COX4I1 | -0.579 | 3.36e-48 |
| COX6A1 | -0.563 | 3.78e-45 |
| COX6C | -0.275 | 1.63e-10 |
| COX7A2 | -0.250 | 6.48e-9 |
| COX7A2L | -0.116 | 7.633e-3 |
| COX7B | -0.434 | 1.78e-25 |
| COX7C | -0.332 | 6.44e-15 |
| COX5A | -0.367 | 4.05e-18 |
| COX5B | -0.502 | 8.79e-35 |
| COX8A | -0.538 | 1.21e-40 |
| COX14 | -0.470 | 3.89e-30 |
| COX17 | -0.271 | 2.92e-10 |
| COX16 | -0.118 | 6.993e-3 |
| COX6B2 | -0.123 | 4.693e-3 |
| COX7A2L | -0.116 | 7.633e-3 |
| ATPAF2 | -0.315 | 1.43e-13 |
| ATP5F1E | -0.222 | 2.67e-7 |
| ATP5MPL | -0.340 | 1.32e-15 |
| ATP5MD | -0.240 | 2.63e-8 |
| ATP5ME | -0.546 | 5.01e-42 |
| ATP5MC2 | -0.414 | 4.08e-23 |
| ATP5F1EP2 | -0.222 | 2.78e-7 |
| ATP5MF | -0.374 | 8.28e-19 |
| ATP5F1D | -0.548 | 2.14e-42 |
| ATP5PD | -0.283 | 4.02e-11 |
| ATP5MG | -0.412 | 6.95e-23 |
| ATP5F1B | -0.150 | 5.950e-4 |
| MRPL12 | -0.512 | 2.31e-36 |
| MRPL52 | -0.280 | 7.10e-11 |
| MRPL27 | -0.539 | 7.53e-41 |
| MRPL23 | -0.565 | 1.57e-45 |
| MRPL47 | -0.0676 | 0.122 |
| MRPL17 | -0.465 | 1.99e-29 |
| MRPS15 | -0.0963 | 0.0275 |
| MRPL14 | -0.462 | 4.42e-29 |
| MRPS11 | -0.321 | 4.97e-14 |
| MRPS26 | -0.354 | 6.34e-17 |
| MRPS24 | -0.527 | 8.71e-39 |
| MRPL53 | -0.409 | 1.62e-22 |
| MRPL28 | -0.456 | 2.61e-28 |
| MRPL51 | -0.179 | 3.817e-5 |
| MRPL21 | -0.423 | 3.45e-24 |
| MRPS26 | -0.354 | 6.34e-17 |
| MRPL38 | -0.493 | 2.19e-33 |
| MRPS12 | -0.400 | 1.55e-21 |
| MRPL22 | -0.236 | 4.59e-8 |
| TOMM40 | -0.432 | 3.22e-25 |
| TOMM6 | -0.275 | 1.43e-10 |
| TOMM7 | -0.279 | 8.23e-11 |
| TOMM5 | -0.211 | 1.109e-6 |
| TOMM22 | -0.232 | 7.95e-8 |
| TOMM34 | -0.145 | 8.929e-4 |
| TOMM20L | -0.162 | 1.884e-4 |
| TIMM22 | -0.0707 | 0.106 |
| TIMM13 | -0.566 | 9.42e-46 |
| TIMM50 | -0.355 | 4.79e-17 |
| TIMM10 | -0.411 | 8.40e-23 |
| TIMM17B | -0.435 | 1.28e-25 |
| TIMM9 | -0.0957 | 0.0284 |
| TIMM44 | -0.474 | 9.71e-31 |
| TIMM29 | -0.267 | 5.39e-10 |
| TIMM8A | -0.0861 | 0.0488 |
| CYC1 | -0.435 | 1.17e-25 |
| SIRT3 | -0.356 | 3.89e-17 |

### Supplementary Table S3 TCGA databases analyses of the correlation between the mRNA expression levels of ZNF281 and mitochondrial related genes in Lung Adenocarcinoma (TCGA, PanCancer Atlas)

| Mitochondrial related genes | Correlation with ZNF281  (Spearman’s correlation coefficient) | *p*-Value |
| --- | --- | --- |
| NDUFS3 | -0.260 | 3.10e-9 |
| NDUFS4 | -0.00299 | 0.947 |
| NDUFS5 | -0.133 | 2.760e-3 |
| NDUFS6 | -0.163 | 3.12e-27 |
| NDUFS7 | -0.440 | 3.20e-25 |
| NDUFS8 | -0.220 | 6.32e-7 |
| NDUFV1 | -0.179 | 5.560e-5 |
| NDUFV3 | -0.103 | 0.0213 |
| NDUFA12 | -0.0966 | 0.0303 |
| NDUFA13 | -0.318 | 2.56e-13 |
| NDUFA4 | -0.0691 | 0.122 |
| NDUFAF4 | -0.0935 | 0.0361 |
| NDUFA11 | -0.377 | 1.85e-18 |
| NDUFAB1 | -0.0991 | 0.0262 |
| NDUFAF8 | -0.193 | 1.286e-5 |
| NUDFA3 | -0.259 | 3.66e-9 |
| NDUFA2 | -0.239 | 5.48e-8 |
| NDUFAF3 | -0.296 | 1.34e-11 |
| NDUFA7 | -0.259 | 3.69e-9 |
| NDUFAF2 | -0.0997 | 0.0253 |
| NDUFA8 | -0.166 | 1.826e-4 |
| NDUFA6 | -0.208 | 2.565e-6 |
| NDUFA1 | -0.277 | 2.58e-10 |
| SDHB | -0.165 | 1.978e-4 |
| SDHAF1 | -0.274 | 4.18e-10 |
| SDHAF2 | -0.0830 | 0.0627 |
| BCS1L | -0.277 | 2.67e-10 |
| UQCRB | -0.116 | 9.383e-3 |
| UQCRQ | -0.217 | 9.24e-7 |
| UQCRC1 | -0.248 | 1.85e-8 |
| UQCRH | -0.0838 | 0.0602 |
| UQCRFS1 | -0.0433 | 0.333 |
| UQCR10 | -0.223 | 4.37e-7 |
| UQCR11 | -0.312 | 7.62e-13 |
| UQCC3 | -0.218 | 7.67e-7 |
| UQCRHL | -0.0134 | 0.764 |
| UQCC2 | -0.186 | 2.565e-5 |
| SURF1 | -0.243 | 3.32e-8 |
| SCO2 | -0.452 | 1.10e-26 |
| COX6B1 | -0.232 | 1.36e-7 |
| COX4I1 | -0.325 | 7.43e-14 |
| COX6A1 | -0.181 | 4.525e-5 |
| COX6C | -0.148 | 8.981e-4 |
| COX7A2 | -0.122 | 6.305e-3 |
| COX7B | -0.321 | 1.63e-13 |
| COX7C | -0.183 | 3.822e-5 |
| COX5A | -0.156 | 4.303e-4 |
| COX5B | -0.201 | 5.655e-6 |
| COX8A | -0.243 | 3.30e-8 |
| COX14 | -0.156 | 4.544e-4 |
| COX17 | -0.238 | 6.35e-8 |
| COX16 | -0.131 | 3.322e-3 |
| COX4I2 | -0.141 | 1.538e-3 |
| COX7A1 | -0.197 | 8.108e-6 |
| COX6B2 | -0.162 | 2.672e-4 |
| ATPAF2 | -0.131 | 3.181e-3 |
| ATP5F1E | -0.229 | 2.13e-7 |
| ATP5MPL | -0.182 | 4.184e-5 |
| ATP5MD | -0.103 | 0.0210 |
| ATP5ME | -0.235 | 9.63e-8 |
| ATP5MC2 | -0.170 | 1.326e-4 |
| ATP5F1EP2 | -0.0523 | 0.242 |
| ATP5MF | -0.196 | 9.964e-6 |
| ATP5F1D | -0.365 | 2.61e-17 |
| ATP5PD | -0.181 | 4.412e-5 |
| ATP5MG | -0.179 | 5.503e-5 |
| ATP5F1B | -0.0585 | 0.208 |
| MRPL12 | -0.178 | 5.998e-5 |
| MRPL52 | -0.270 | 8.03e-10 |
| MRPL27 | -0.227 | 2.73e-7 |
| MRPL23 | -0.231 | 1.67e-7 |
| MRPL47 | -0.148 | 8.628e-4 |
| MRPL17 | -0.211 | 1.756e-6 |
| MRPS15 | -0.109 | 0.0142 |
| MRPL14 | -0.164 | 2.222e-4 |
| MRPS11 | -0.157 | 3.972e-4 |
| MRPS26 | -0.0556 | 0.213 |
| MRPS24 | -0.179 | 5.329e-5 |
| MRPL53 | -0.157 | 4.181e-4 |
| MRPL28 | -0.187 | 2.364e-5 |
| MRPL51 | -0.120 | 7.026e-3 |
| MRPL21 | -0.194 | 1.151e-5 |
| MRPS26 | -0.0556 | 0.213 |
| MRPL38 | -0.138 | 1.905e-3 |
| MRPS12 | -0.153 | 5.893e-4 |
| MRPL22 | -0.135 | 2.342e-3 |
| TOMM40 | -0.0596 | 0.182 |
| TOMM6 | -0.0329 | 0.462 |
| TOMM7 | -0.197 | 8.133e-6 |
| TOMM5 | -0.124 | 5.432e-3 |
| TOMM22 | -0.0808 | 0.0701 |
| TOMM34 | -0.161 | 2.971e-4 |
| TOMM20L | -0.0137 | 0.759 |
| TIMM22 | -0.139 | 1.840e-3 |
| TIMM13 | -0.259 | 3.71e-9 |
| TIMM50 | -0.0649 | 0.146 |
| TIMM10 | -0.183 | 3.826e-5 |
| TIMM17B | -0.110 | 0.0139 |
| TIMM9 | -0.0788 | 0.0775 |
| TIMM44 | -0.296 | 1.36e-11 |
| TIMM29 | -0.183 | 3.493e-5 |
| TIMMDC1 | -0.230 | 1.90e-7 |
| TIMM8A | -0.0423 | 0.343 |
| CYC1 | -0.103 | 0.0214 |
| SIRT3 | -0.114 | 0.0105 |
