# Supplementary material for: ZNF281 inhibits mitochondrial biogenesis to facilitate metastasis of hepatocellular carcinoma
Source: Cell Death Discov. 2023 Oct 25;9:396. doi: 10.1038/s41420-023-01691-9 (PMC10600106; doi:10.1038/s41420-023-01691-9)
Supplement: Supplementary file 2 — Full and uncropped western blots [file 41420_2023_1691_MOESM2_ESM.pptx]

## Slide 1
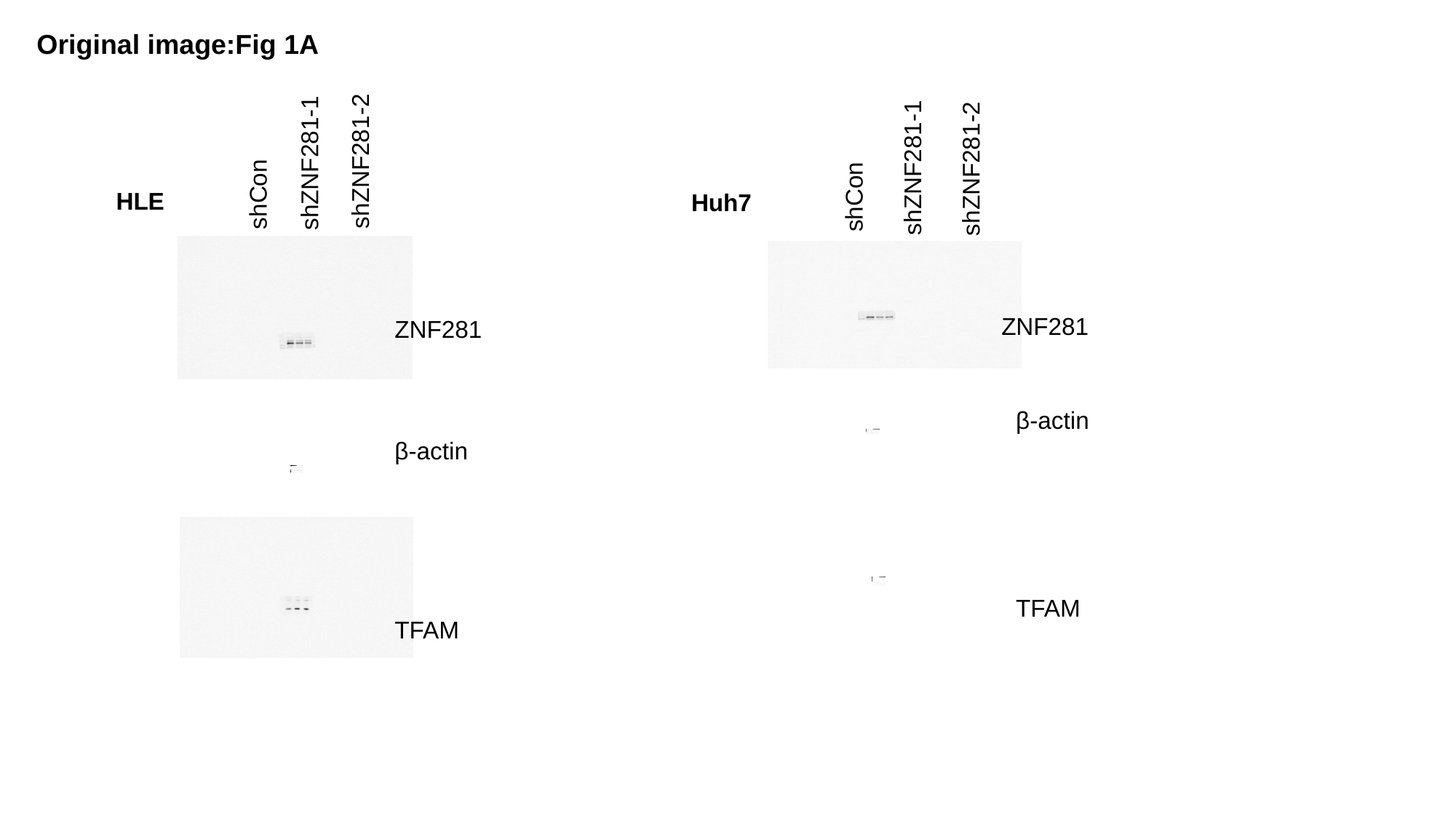

Original image:Fig 1A
shZNF281-2
shZNF281-1
shCon
HLE
ZNF281
β-actin
TFAM
shZNF281-1
shZNF281-2
shCon
Huh7
ZNF281
β-actin
TFAM

## Slide 2
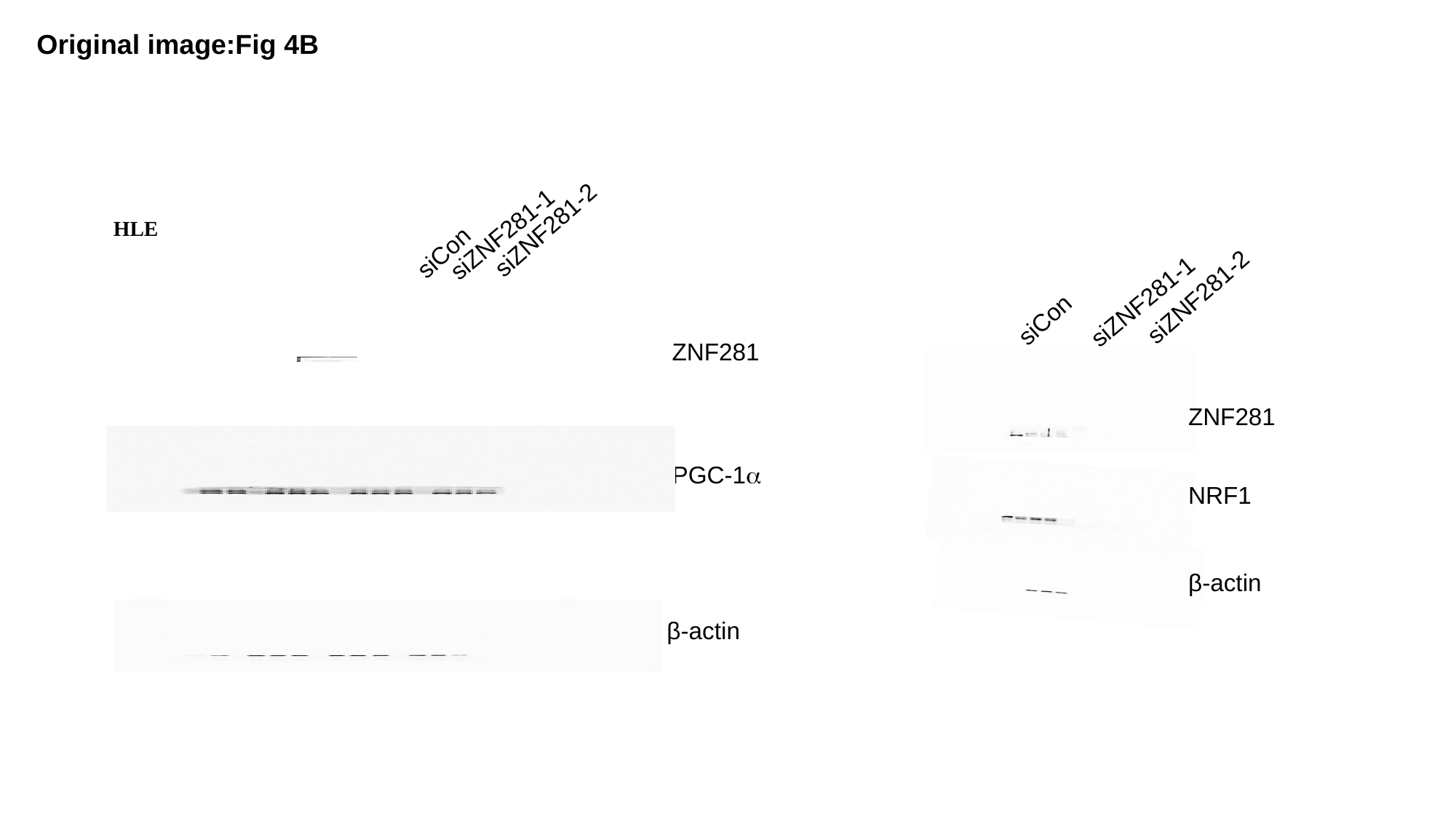

Original image:Fig 4B
siZNF281-2
siZNF281-1
HLE
siCon
ZNF281
PGC-1α
β-actin
siZNF281-2
siZNF281-1
siCon
ZNF281
NRF1
β-actin

## Slide 3
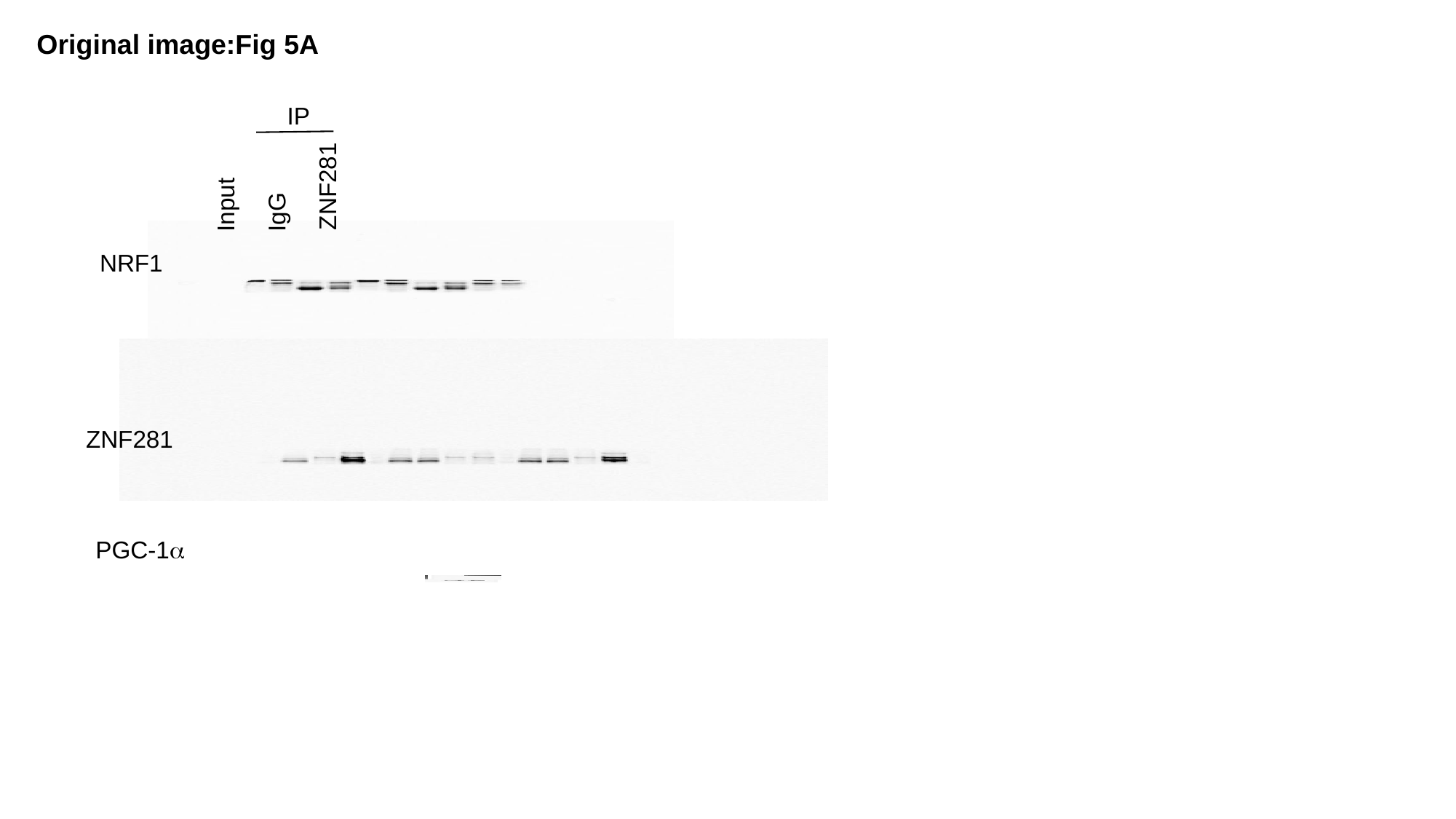

Original image:Fig 5A
IP
ZNF281
Input
IgG
NRF1
ZNF281
PGC-1α

## Slide 4
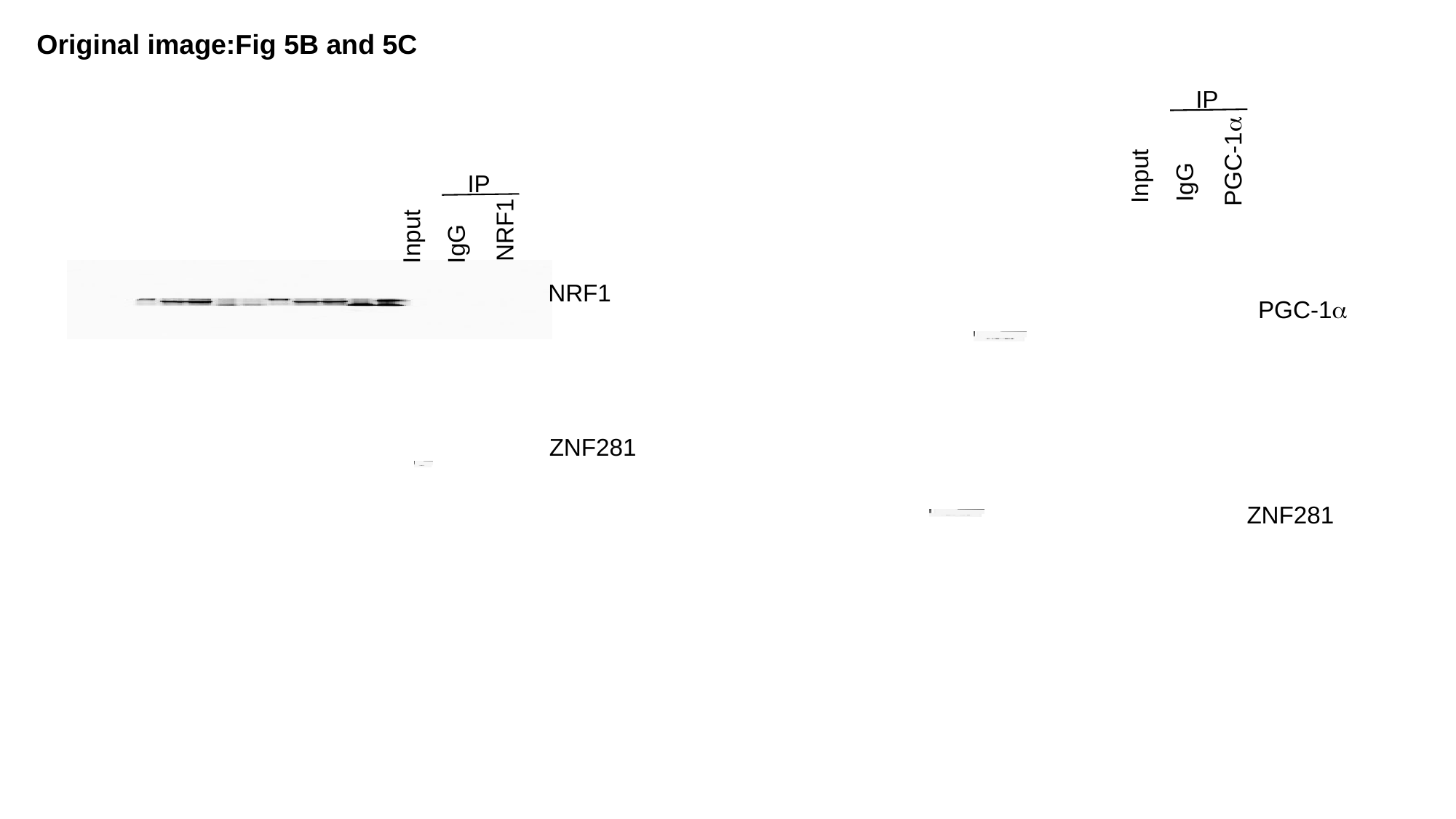

Original image:Fig 5B and 5C
IP
PGC-1α
IgG
Input
PGC-1α
ZNF281
IP
Input
IgG
NRF1
NRF1
ZNF281

## Slide 5
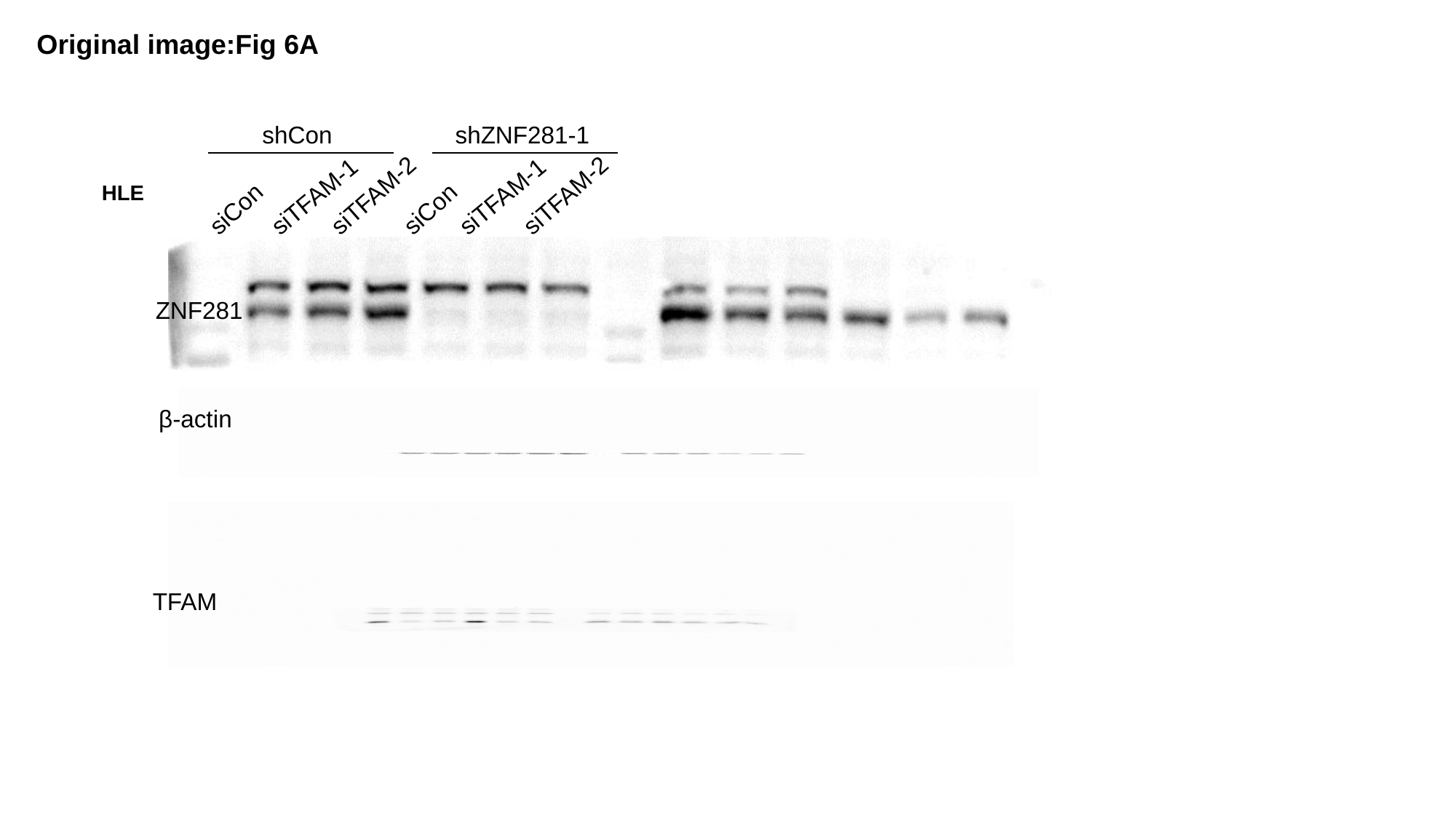

Original image:Fig 6A
shCon
shZNF281-1
siTFAM-2
siTFAM-2
HLE
siCon
siCon
ZNF281
β-actin
TFAM
siTFAM-1
siTFAM-1

## Slide 6
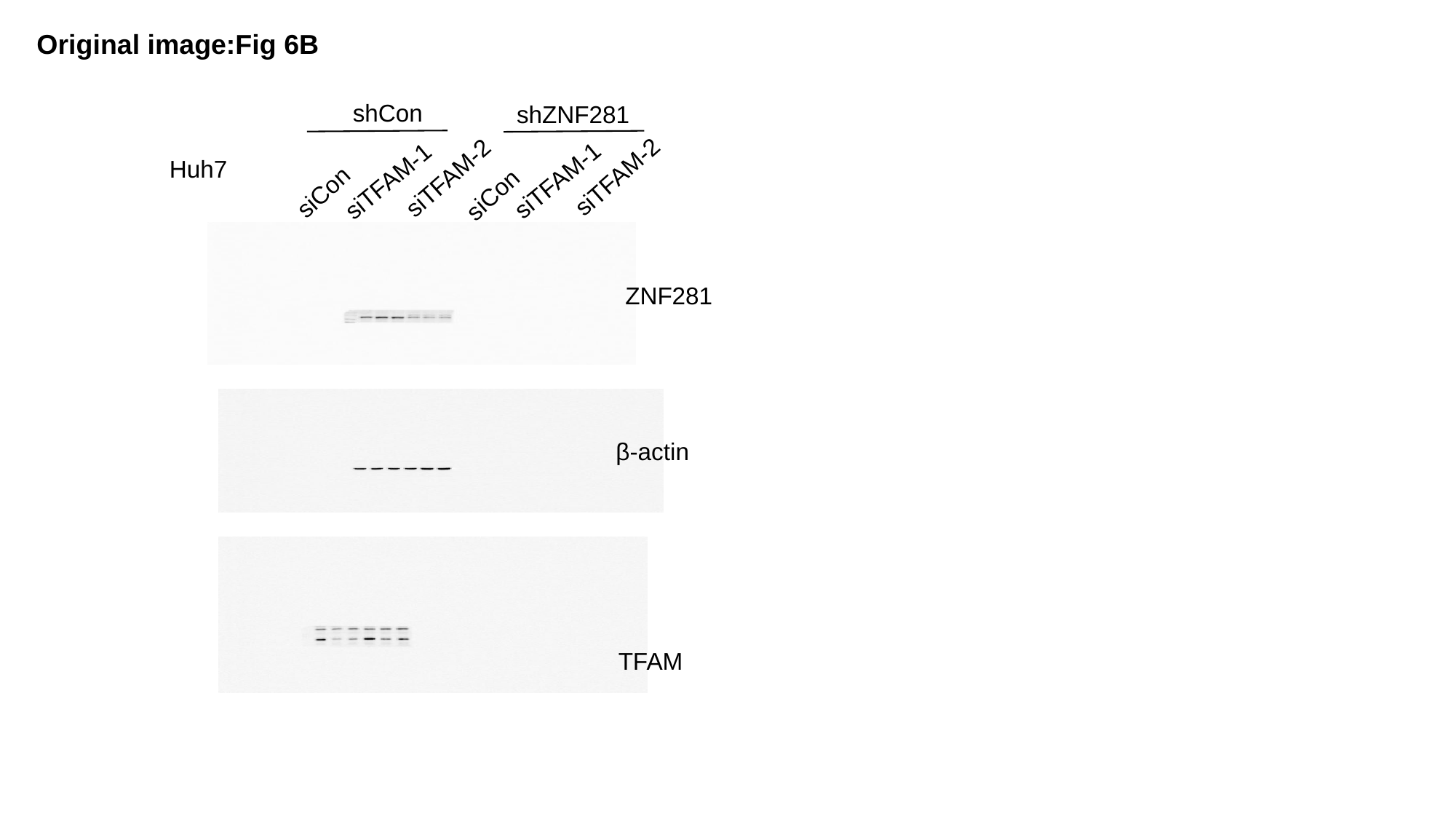

Original image:Fig 6B
shCon
shZNF281
Huh7
ZNF281
β-actin
TFAM
siTFAM-2
siTFAM-1
siTFAM-2
siTFAM-1
siCon
siCon

## Slide 7
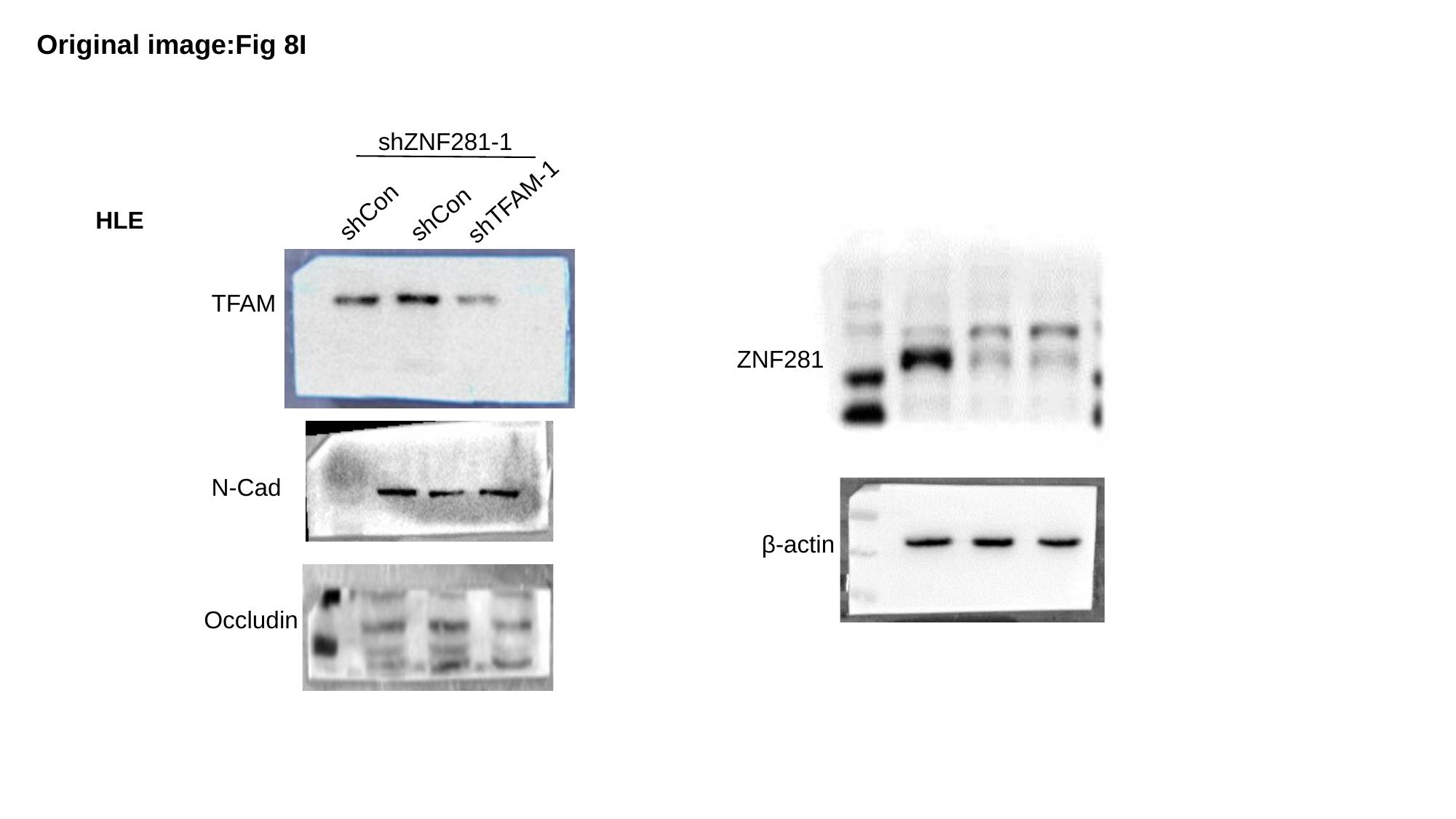

Original image:Fig 8I
shZNF281-1
shTFAM-1
shCon
shCon
HLE
TFAM
ZNF281
N-Cad
β-actin
Occludin

## Slide 8
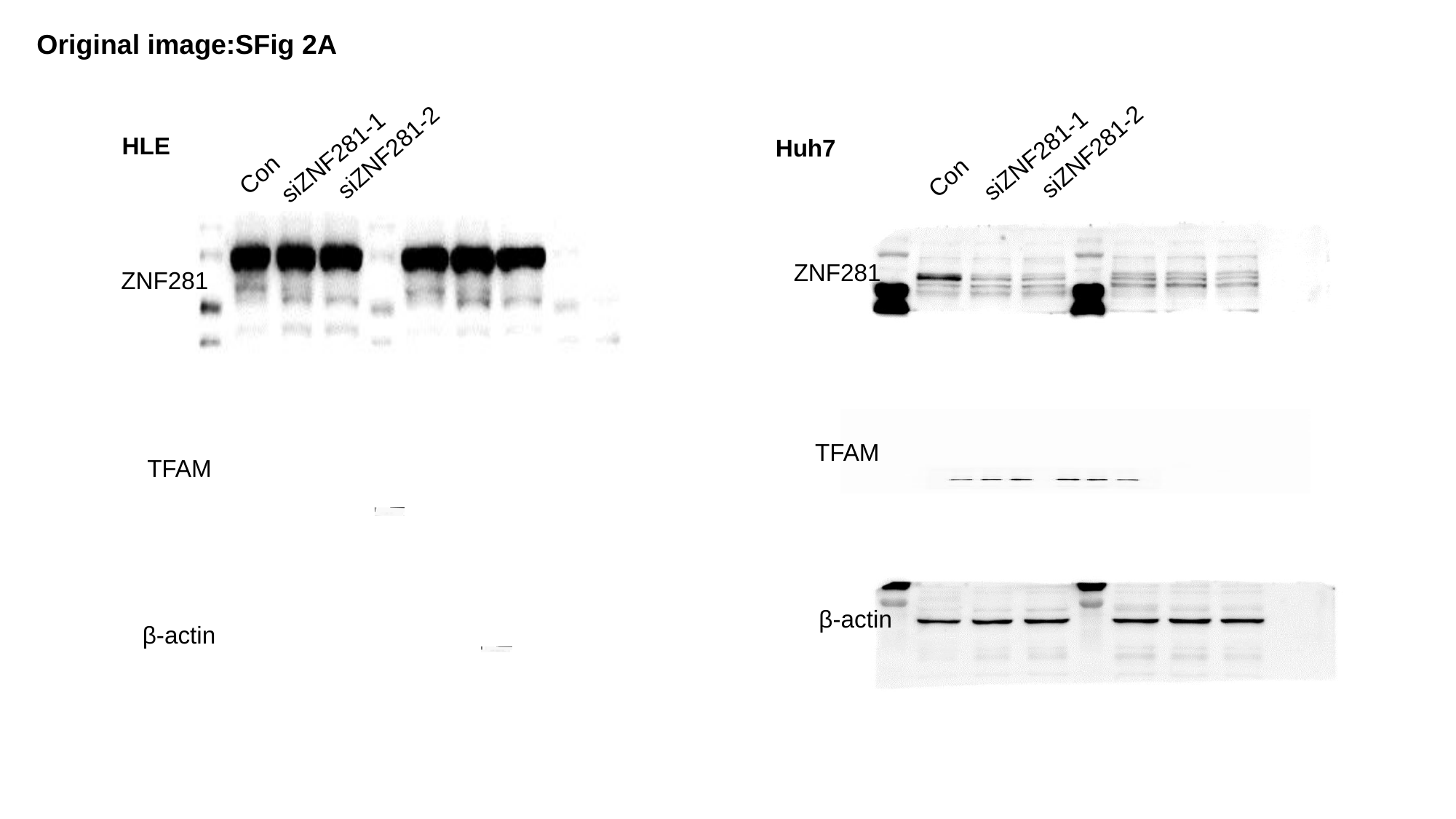

Original image:SFig 2A
siZNF281-2
siZNF281-1
Huh7
Con
ZNF281
TFAM
β-actin
HLE
Con
ZNF281
TFAM
β-actin
siZNF281-2
siZNF281-1

## Slide 9
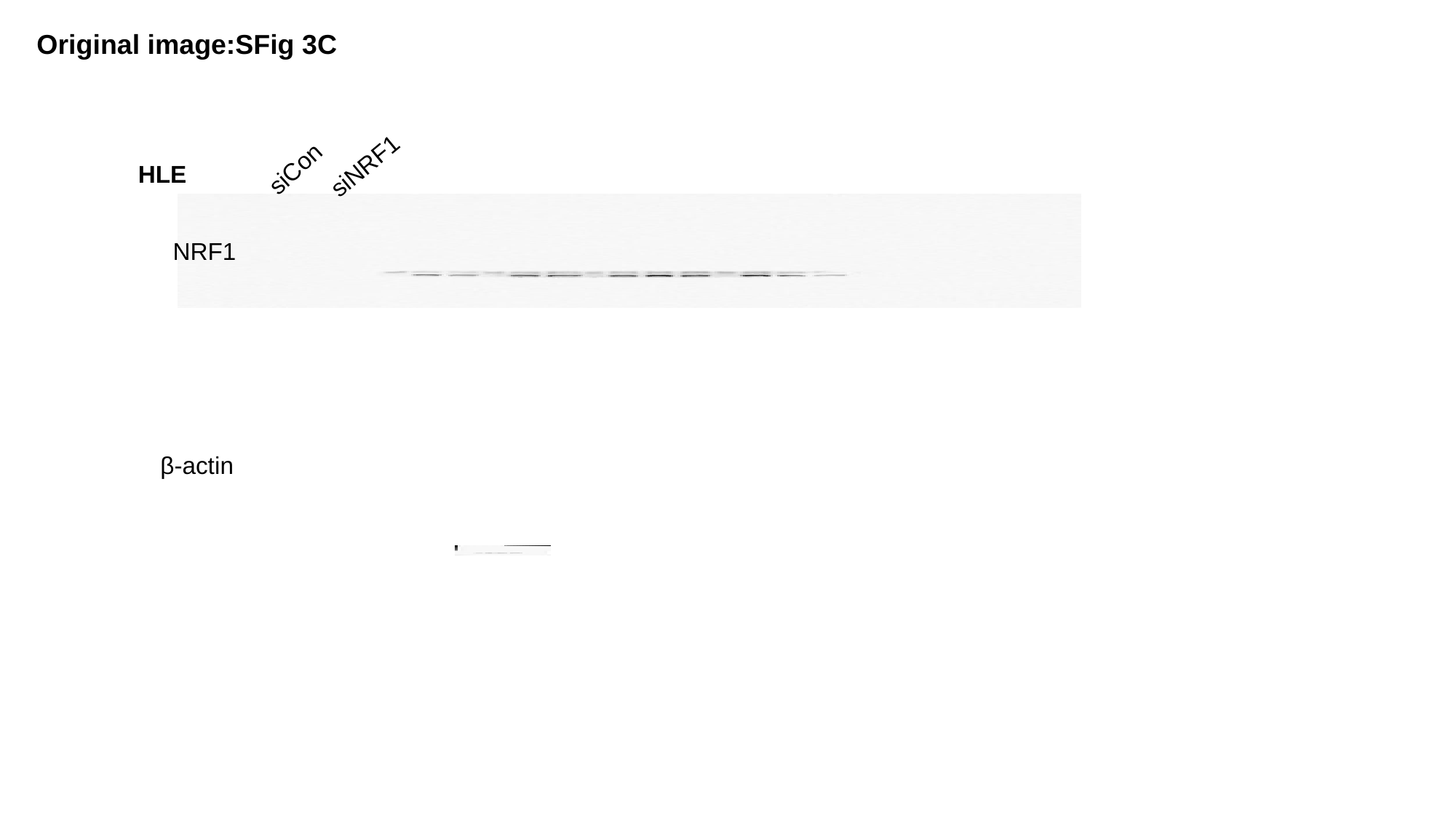

Original image:SFig 3C
siNRF1
siCon
HLE
NRF1
β-actin
